# MSI: A Mahalanobis‐Based Molecular Similarity Index for High‐Dimensional Embeddings

**DOI:** 10.1002/minf.70051

**Published:** 2026-08-27

**Authors:** Roberto Bernal‐Jaquez, Elliot Ridout‐Buhl, Emiliano Montoya, León Alday‐Toledo, Felipe Aparicio

**Affiliations:** ^1^ Departamento de Matemáticas Aplicadas y Sistemas Universidad Autónoma Metropolitana Unidad Cuajimalpa Ciudad de México México; ^2^ Departamento de Ciencias Naturales Universidad Autónoma Metropolitana Unidad Cuajimalpa Ciudad de México México; ^3^ Posgrado en Ciencias Naturales e Ingeniería Universidad Autónoma Metropolitana Unidad Cuajimalpa Ciudad de México México

**Keywords:** angular similarity, high‐dimensional chemical space, Mahalanobis distance, Mahalanobis Similarity Index (MSI), molecular similarity

## Abstract

Quantifying molecular similarity is crucial for drug discovery and for exploring chemical space. A similarity assessment always combines two independent ingredients: a molecular representation and a similarity coefficient. The most common pairing, binary substructure fingerprints scored with the Tanimoto coefficient, depends strongly on fingerprint bit density and, because it compares unweighted *sets* of substructure identifiers, is blind to the multiplicity of repeated fragments and frequently returns ranking ties that obscure meaningful chemical relationships. Here, we introduce the Mahalanobis Similarity Index (MSI), which pairs continuous Mol2Vec embeddings with a covariance‐aware Mahalanobis distance (*d*_M_) and an associated Mahalanobis angle (*θ*_M_) to give a statistically grounded assessment that is invariant under invertible linear reparametrization of the descriptor space. We evaluated MSI on five chemically distinct reference compounds: aspirin, a salicylate nonsteroidal anti‐inflammatory drug (NSAID); aniline, an industrial aromatic amine; curcumin, a polyphenolic natural product; ibuprofen, a propionic‐acid NSAID; and digitoxin, a cardiac glycoside. Relative to the Tanimoto coefficient computed on ECFP4 fingerprints, MSI improves the analysis in three specific respects: it promotes chemically reasonable analogs that the fingerprint deprioritises; it resolves ranking ties, recovering between 7 and 10 distinct scores among the 10 nearest neighbors where Tanimoto recovers only 3–7; and its geometry varies systematically with HOMO–LUMO energy gaps in the QM9 dataset, indicating that the embedding tracks electronic structure even though it was trained on structural context alone. Polar plots and three‐dimensional similarity maps reveal anisotropy within the embedding space and define practical applicability domains for high‐similarity retrieval. MSI retains discriminatory power in the regime where the Tanimoto coefficient saturates near zero, and sparse peripheral regions suggest scaffold‐hopping opportunities. The dual radial–angular description supports hypothesis‐driven reasoning about how structural modifications shift electronic properties. MSI is computationally efficient, chemically interpretable, and offers a practical way to navigate high‐dimensional chemical space. Beyond drug discovery, it is applicable to materials science, toxicology, and chemical biology, wherever continuous molecular embeddings are used for property‐driven screening.

## Graphical abstract

MSI pairs continuous Mol2Vec embeddings with a covariance‐aware Mahalanobis metric (*d*_M_, *θ*_M_) to overcome the ranking ties and bit‐density bias of Tanimoto fingerprints. Across five reference compounds, MSI resolves more distinct neighbors than ECFP4 + Tanimoto and its geometry tracks HOMO–LUMO gaps in QM9, despite no electronic training.

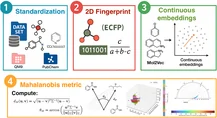

**Abbreviations**
MSI: Mahalanobis Similarity Index
TSI: Tanimoto Similarity Index

## 1 Introduction

Molecular similarity assessment sits at the core of modern computational chemistry and drug discovery, offering a quantitative framework for comparing chemical structures and predicting their biological activity [1, 2, 3]. The underlying premise is the *Similar Property Principle*: structurally similar molecules tend to exhibit similar physicochemical properties and biological activities [4, 5]. This principle has guided numerous applications in drug discovery, including virtual screening [6], lead optimization [5], and the design of compound libraries [7].

The quantification of molecular similarity relies on mathematical indices that translate the structural features of the molecules into numerical values, enabling systematic comparison across chemical spaces. These indices have evolved significantly over the past decades, from simple fragment‐based approaches to sophisticated machine learning algorithms, including three‐dimensional structural information and pharmacophoric features [8]. The selection of an appropriate similarity measure depends on the specific context and objectives of the analysis, as different indices emphasize different aspects of molecular structure [9, 10, 11].

A precise treatment of molecular similarity requires a clear distinction between its two independent components: (i) the *molecular representation*, which encodes structural and chemical information as a numerical vector (e.g., binary fingerprints, continuous physicochemical descriptors, or learned embeddings), and (ii) the *similarity coefficient* (or metric), a scalar or a distance that quantifies the relationship between two such vectors [1, 2, 12]. This distinction is critical: the Tanimoto coefficient, for instance, is not inherently tied to fingerprint representations and can be applied to any suitably normalized descriptor space, including continuous ones [8]. Conversely, the Mahalanobis distance is not tied to embeddings and can be evaluated on any real‐valued descriptor vector. Throughout the present work we therefore name representation and coefficient separately: the baseline against which MSI is compared is *ECFP4 + Tanimoto*, not “Tanimoto similarity” as such, and MSI itself is *Mol2Vec + Mahalanobis*.

Traditional similarity searches, which pair binary substructure fingerprints with the Tanimoto coefficient (also known as the Jaccard index), remain widely used because of their computational efficiency and intuitive interpretation [13, 14]. These approaches represent molecules as binary vectors, indicating the presence or absence of predefined structural features, with similarity calculated from the overlap between these vectors. Within chemoinformatics, the Tanimoto coefficient—calculated as the proportion of features jointly possessed by two molecules to the total features present in either molecule—has emerged as the de facto benchmark for measuring molecular similarity [8, 15, 16].

More recent developments in molecular similarity assessment include graph‐based methods [17, 18], which preserve the topological relationships between atoms: pharmacophore‐based approaches [19], which focus on the spatial arrangement of functional groups, and molecular field‐based techniques [20], which compare the three‐dimensional distribution of electronic and steric properties. These methods provide complementary perspectives on molecular similarity, capturing aspects that fingerprint‐based indices may overlook.

Machine learning has further revolutionized the evaluation of molecular similarity, enabling the development of data‐driven similarity metrics to capture the complex and nonlinear relationships between molecular structure and biological activity [21, 22, 23]. In particular, Mol2Vec [24] adapts natural language word embedding strategies to chemistry, like Word2Vec [25, 26], producing continuous vectors for molecular fragments that encode contextual information unavailable to fixed‐length fingerprints.

Although researchers commonly rely on Euclidean distance for learning molecular representations, this conventional approach overlooks correlations between descriptor dimensions, a real limitation for chemical space analysis. The Mahalanobis distance (*d*_M_), introduced by P. C. Mahalanobis [27] in 1936, addresses this shortcoming by incorporating the inverse covariance matrix to scale each dimension appropriately. The metric has since found use across a range of scientific applications, from multivariate quality control to cryoelectron microscopy [28]. Applying *d*_M_ to chemical embeddings gives us a principled way to correct for the anisotropy inherent to high‐dimensional chemical space.

In this paper, we introduce the Mahalanobis Similarity Index (MSI), which couples Mol2Vec embeddings with the Mahalanobis framework to generate both a covariance‐aware distance and an angle (*θ*_M_) between molecular vectors. Because the covariance matrix incorporates interdescriptor correlations, MSI mitigates size bias and faithfully represents the angular and radial relationships among molecules. We compare MSI against the ECFP4 + Tanimoto baseline using five reference compounds distributed over three retrieval testbeds: (i) aspirin, curcumin, and ibuprofen, queried against a library of aspirinoid–curcuminoid–ibuprofenoid (ACI) molecules obtained from PubChem [29]; (ii) aniline, queried against the QM9 database [30]; and (iii) digitoxin, queried against the ACI library augmented with digitoxin‐like molecules from PubChem. Across all three testbeds MSI produces markedly fewer ranking ties and chemically more interpretable neighborhoods than the fingerprint‐based search. We emphasize that these are retrieval and ranking comparisons on curated analog libraries; no prospective virtual‐screening benchmark with known actives and decoys is attempted here, so no enrichment factors are reported (see Limitations, Section 4.6).

The term *chemical space* is used in several distinct senses in the literature, and we therefore state ours explicitly. Throughout this work, *chemical space* denotes the abstract, high‐dimensional metric space in which every molecule is represented by a single vector (or other suitable mathematical object) together with a metric that makes distances between such objects meaningful [31]. The number of conceivable organic molecules is enormous—roughly 10^60^ structures for compounds of up to 30 heavy atoms [32], and about 10^33^ when the count is restricted to drug‐like molecules obeying standard property filters [33]—so any practical analysis is necessarily restricted to a sampled subset, and every result reported here is conditional on the library that was sampled. We further distinguish this space, which is the object of study, from its *visual representations*: the scatter plots, polar maps, and three‐dimensional surfaces shown below are two‐ or three‐dimensional summaries of selected coordinates and are intended to aid interpretation, not to render the full 300‐dimensional embedding faithfully. The specific space explored in this work is defined by the 300‐dimensional Mol2Vec vectors endowed with the Mahalanobis metric, and we probe it through the pair of coordinates (*d*_M_, *θ*_M_) computed with respect to a chosen query molecule.

By combining continuous molecular representations with the statistically rigorous Mahalanobis metric, MSI addresses three long‐standing challenges in drug discovery and medicinal chemistry: similarity searching, scaffold hopping, and the visualization of expansive chemical spaces.

## 2 Theoretical Framework

### 2.1 Advanced Molecular Representation Techniques

#### 2.1.1 Mol2Vec Embedding Methodology

Traditional molecular fingerprinting approaches rely on fixed‐length descriptors that capture structural features but often fail to represent complex contextual relationships between molecular substructures. To address these limitations, contemporary machine learning approaches have adapted natural language processing (NLP) methodologies to create more accurate molecular representations. The Mol2Vec algorithm represents a significant advancement in this domain, adapting the Word2Vec framework from computational linguistics to generate continuous vector embeddings of molecular substructures [24].

The implementation of Mol2Vec follows a systematic two‐phase methodology:

1. **Substructure identification and extraction**: Molecules are decomposed into circular overlapping substructures using the Morgan algorithm [34], generating structural “vocabulary” elements that serve as the fundamental units for subsequent embedding.
2. **Contextual vector embedding generation**: Borrowing the Word2Vec neural framework, Mol2Vec learns a vector $v_{i}\in R^{300}$ for every Morgan identifier *i* by maximizing the likelihood of the identifiers that co‐occur with *i* in the same molecule. In the continuous bag‐of‐words formulation, the context used to predict the target identifier *i* is summarized as
(1) $$c_{i}=\frac{1}{\left|N(i)\right|}\sum_{j\in N(i)}w_{j}$$
  where N(i) denotes the set of substructure identifiers that occur in the neighborhood of *i* and **w**_*j*_ is the input (context) vector of identifier *j*; the learned output vectors **v**_*i*_ are the substructure embeddings used subsequently.
3. **Molecule vector assembly**: The vector of a whole molecule *M* is obtained as the *sum* of the embeddings of all Morgan identifiers it contains, counted with multiplicity:
(2) $$x_{M}=\sum_{i\in M}m_{i}\;v_{i}$$
  where *m*_*i*_ is the number of times identifier *i* occurs in *M*. This point is not incidental for the present work: because the sum in Equation (2) is unnormalized and multiplicity‐weighted, the Mol2Vec representation retains information about *how many* copies of a fragment a molecule contains, whereas a binary fingerprint—which records only whether an identifier is present—does not. Several of the discrepancies between MSI and Tanimoto rankings reported in Section 4 trace directly to this difference.

The resulting continuous vector representations preserve relational information between molecular substructures, enabling similarity analyses that capture structural relationships beyond simple fingerprint‐based matching. Two limitations of the pretrained Mol2Vec model used here should be stated at the outset. First, the Morgan identifiers are generated without chirality flags, so the embedding is blind to stereochemistry: two stereoisomers receive identical vectors and hence their *d*_M_ is the same. Second, any molecule containing a fragment absent from the model vocabulary cannot be embedded and is discarded (Section 3).

It is well established in the chemoinformatics literature that similarity outcomes depend critically on the chosen molecular representation, often to a greater extent than on the similarity coefficient itself [2, 12]. The choice of representation determines which structural and physicochemical features are encoded and compared, fundamentally shaping the resulting similarity landscape. In the MSI framework, the Mol2Vec embedding and the Mahalanobis metric are not independent choices: the metric is specifically designed to exploit the covariance structure inherent to the embedding space. This coupling is a deliberate design feature—the Mahalanobis correction without a continuous embedding would be uninformative. Conversely, a Mol2Vec embedding evaluated with Euclidean distance would discard the interdescriptor correlations that Mol2Vec encodes. The inseparability of representation and metric in MSI thus reflects a unified statistical framework rather than a limitation, and any fair comparison must account for both components simultaneously.

### 2.2 Mahalanobis‐Based Similarity Frameworks

#### 2.2.1 Mahalanobis Distance Metric

The Mahalanobis distance (*d*_M_) provides a sophisticated multivariate metric that accounts for correlations within descriptor spaces. Unlike conventional Euclidean distance measures, which treat variables as independent, *d*_M_ incorporates the covariance structure of the data, creating a scale‐invariant distance measure particularly valuable for correlated molecular descriptors. The formal mathematical definition of *d*_M_ between vectors **u** and **v** relative to a covariance matrix Σ is

(3) $$d_{M}(u,v)=\sqrt{{\left(u-v\right)}^{T}\Sigma^{-1}(u-v)}$$

where Σ^−1^ is the inverse of the covariance matrix, estimated here over the full library of embedded molecules. Equation (3) is equivalent to the ordinary Euclidean distance computed in *whitened* coordinates $x=\Sigma^{-1/2}u$, and it defines a genuine metric provided Σ is positive definite. The resulting quantity is dimensionless and is invariant under any invertible linear reparametrization of the descriptor space. We refer to this property as *scale invariance* throughout this work, and it should not be confused with invariance to molecular size. Incorporating covariance information makes *d*_M_ particularly suitable for molecular similarity calculations in high‐dimensional spaces where descriptor correlations significantly influence similarity determinations.

Within this framework, *d*_M_ exploits the covariance structure of the Mol2Vec embedding space, downweighting redundant, strongly correlated directions and giving comparatively more weight to directions of low variance.

#### 2.2.2 Mahalanobis Angular Similarity Measure

To further enhance similarity quantification, we introduce the Mahalanobis angle (*θ*_M_) as a complementary similarity measure that captures both directional and scalar relationships between molecular vectors. The *θ*_M_ measure evaluates the angular separation between vectors in a normalized Mahalanobis space, providing a similarity measure that accounts for molecular feature correlations while normalizing vector magnitudes.

For molecular vectors **u** and **v** within the Mol2Vec embedding space, *θ*_M_ is calculated using the inverse covariance matrix Σ^−1^ as follows:

(4) $$\cos\;(\theta_{M})=\frac{u^{T}\Sigma^{-1}v}{\|u{\|}_{\Sigma }\|v{\|}_{\Sigma }}$$

where $u^{T}\Sigma^{-1}v$ represents the Mahalanobis inner product, and $\|u{\|}_{\Sigma }=\sqrt{u^{T}\Sigma^{-1}u}$ and $\|v{\|}_{\Sigma }=\sqrt{v^{T}\Sigma^{-1}v}$ denote the Mahalanobis norms of vectors **u** and **v**, respectively.

The angular measurement *θ*_M_ is then derived as

(5) $$\theta_{M}=\arccos\;\left(\frac{u^{T}\Sigma^{-1}v}{\|u{\|}_{\Sigma }\|v{\|}_{\Sigma }}\right)$$

Smaller angular values indicate closer alignment of the two molecular vectors in whitened space, and *θ*_M_ is bounded in [0°, 180°] irrespective of dimensionality, which makes it convenient for setting thresholds that transfer between libraries of different size.

Two properties of *θ*_M_ deserve explicit statement because they bound what the angular coordinate can contribute. First, *θ*_M_ is computed between the position vectors of the two molecules measured from the *origin of the embedding space*, not from the query molecule; only *d*_M_ is referred to the query. The pair (*d*_M_, *θ*_M_) is therefore a mixed, rather than a strictly polar, coordinate system. Second, in whitened coordinates $x=\Sigma^{-1/2}u$ and $y=\Sigma^{-1/2}v$ the two quantities are linked by the law of cosines:

(6) $${d_{M}}^{2}=\|x{\|}^{2}+\|y{\|}^{2}-2\;\|x\|\;\|y\|\cos\;\theta_{M}$$

Whenever the whitened norms ‖y‖ are approximately constant across a library—as concentration of measure makes likely in 300 dimensions—*d*_M_ becomes a monotone function of *θ*_M_ for a fixed query. This is precisely what we observe: the Spearman rank correlation between *d*_M_ and *θ*_M_ is *ρ *= 1.0000 for every library examined (Table 1). The angular coordinate should therefore be understood as a bounded, scale‐compressed re‐expression of the radial coordinate rather than as an independent source of discrimination, and we present it as such below; its practical value lies in providing dimension‐insensitive thresholds, not in resolving molecules that *d*_M_ cannot.

**TABLE 1:** Cross‐compound consistency of the MSI tie‐breaking advantage.

Reference	*n*	Mean TSI	Mean *d*_M_	Mean *θ*_M_,°	Pearson *r* (TSI, *d*_M_)	Distinct, top‐10 MSI	Distinct, top‐10 TSI
Aspirin	31 393	0.2461	16.77	3.64	−0.2706	7/10	4/10
Curcumin	31 393	0.2001	16.99	3.69	−0.2721	9/10	3/10
Ibuprofen	31 393	0.1829	18.43	4.00	−0.2176	10/10	4/10
Digitoxin	32 903	0.0876	18.19	4.92	−0.1575	8/10	7/10

*Note:* For each reference molecule the table reports library‐wide summary statistics and the number of distinct values found among the 10 nearest neighbors ranked by *d*_M_ versus by TSI. Aspirin, curcumin, and ibuprofen were queried against the same *n* = 31 393‐molecule ACI library; digitoxin was queried against the *n* = 32 903‐molecule library obtained by augmenting the ACI set with digitoxin‐type compounds (Section 4.4.3).

Taken together, these two quantities define the index used throughout this work: MSI denotes the ordered pair (*d*_M_, *θ*_M_) evaluated between a query molecule and a candidate in the Mol2Vec embedding space, with rankings taken on *d*_M_ unless stated otherwise. MSI is thus a dissimilarity descriptor—smaller values mean greater similarity—and is not normalized to the [0, 1] interval of the Tanimoto coefficient.

### 2.3 Molecular Similarity Metrics

#### 2.3.1 Tanimoto Coefficient

The Tanimoto coefficient, widely recognized as the Tanimoto Similarity Index (TSI), represents a cornerstone metric in contemporary cheminformatics for quantifying molecular similarities through fingerprint comparisons [8]. This coefficient quantifies the degree of overlap between molecular feature sets, with particular efficiency for binary fingerprint data. For two molecular fingerprints represented as sets *A* and *B*, the following equation defines the mathematical formulation of the TSI:

(7) $$\mathrm{TSI}(A,B)=\frac{\left|A\cap B\right|}{\left|A|+|B|-|A\cap B\right|}$$

In this expression, |*A*| and |*B*| represent the cardinality of each fingerprint (the count of “on” bits or active features), and |A∩B| denotes the intersection between these feature sets (shared elements). The TSI generates normalized values ranging from 0 to 1, where unity indicates perfect similarity and zero signifies complete dissimilarity. The computational efficiency and intuitive interpretation of the TSI have established it as the predominant metric for high‐throughput similarity searches across extensive molecular databases.

The conventional TSI exhibits inherent limitations, notably a bias favoring smaller molecular structures. To address this constraint, Fligner et al. [35] developed a modified Jaccard–Tanimoto coefficient incorporating size‐adjustment factors. This refinement mitigates the tendency of traditional TSI to overestimate similarities when comparing molecules of substantially different sizes, a critical issue when evaluating diverse chemical libraries. The modification enhances comparative reliability across heterogeneous molecular datasets by introducing normalization factors that account for dimensional disparities [36].

Recent methodological advancements by Miranda‐Quintana et al. [37, 38] have extended similarity assessment beyond binary comparisons by developing extended similarity indices. These novel metrics transcend traditional pairwise evaluations, enabling simultaneous multimolecular comparisons. The capacity for multidimensional similarity analysis significantly enhances chemical space exploration, facilitates more comprehensive diversity assessments, and improves computational throughput in applications ranging from lead optimization to chemical library curation.

A notable generalization of the Tanimoto coefficient is the Tversky index [39], based on the *ratio model* proposed by Tversky, which introduces asymmetric weighting parameters *α* and *β* for the features unique to each molecule:

(8) $$T_{\alpha ,\beta }(A,B)=\frac{\left|A\cap B\right|}{\left|A\cap B|+\alpha |A\setminus B|+\beta |B\setminus A\right|}$$

where A∖B is the set of features in *A* not present in *B*.

The Tanimoto coefficient is recovered when *α *= *β* = 1, and the Dice coefficient when $\alpha =\beta =\frac{1}{2}$; the Tversky index thus embeds both in a one‐parameter family and adds flexibility for directional queries, such as substructure searches in which the query is much smaller than the target. Similar generalizations exist on the side of the representation rather than the coefficient, most notably the extended similarity indices of Miranda‐Quintana et al. [37, 38] discussed above. These extensions broaden the expressiveness of set‐based measures considerably, but they operate on the same underlying object: an unweighted set of substructure identifiers. Consequently, they inherit two properties of that object—the inability to encode correlations between descriptor dimensions and insensitivity to fragment multiplicity—which are the two properties that the continuous, covariance‐weighted formulation of MSI is designed to address. We stress that this is a difference of representation as much as of coefficient and that the Tversky index could itself be applied to a continuous representation, just as the Mahalanobis metric could be applied to a fingerprint.

## 3 Methodology

We implemented all computational analyses using Python 3.8 as the primary environment. For molecular processing, we utilized RDKit 2023.03.1 to work with the Simplified Molecular Input Line Entry System (SMILES) [40, 41] and generate molecular fingerprints. We use Mol2Vec to provide vector embeddings through its pretrained model_300dim.pkl model [24]. The numerical computations leveraged NumPy, SciPy, and Pandas libraries. For data visualization and trend analysis, we employed Matplotlib and Seaborn for plotting and the Locally Weighted Scatterplot Smoothing (LOWESS) [42, 43] routine from statsmodels with a smoothing parameter or frac = 0.5.

All source code, input files, and Jupyter notebooks are available under the MIT license at https://github.com/JACKNINES/MSI; see the Data Availability statement for details.

### 3.1 General Workflow

1. **Standardization**. Our pipeline retrieves SMILES strings from PubChem [29] and QM9 [30], filters out duplicates, and kekulizes the remaining molecules with RDKit. It then generates canonical forms to improve traceability, yet the downstream workflow stays fully compatible with noncanonical representations.
2. **2D fingerprints**. We generate circular extended‐connectivity fingerprints [13] for every molecule. Unless stated otherwise, all Tanimoto values reported in the main text were computed on ECFP4 fingerprints (Morgan radius 2) folded to 2048 bits. The effect of the folding length and of the Morgan radius is examined in Section 4.5.
3. **Continuous embeddings**. We convert each molecule to a 300‐dimensional Mol2Vec vector and discard any molecule that contains fragments outside the model vocabulary, thereby ensuring valid embeddings.
4. **Similarity indices**. We calculate the similarity between the target molecule and the rest of the molecules in the dataset using the Mahalanobis and the Tanimoto indices:
  1. **Mahalanobis indices**. Using all embedded molecules of the library under study, we compute the empirical covariance matrix Σ of the 300‐dimensional embedding space. Because the number of molecules exceeds the dimensionality by two orders of magnitude in every library considered here (*n *≥ 31 393, *p* = 300), Σ is well determined; we nonetheless add a Tikhonov regulariser [44] (*λ *= 10^−5^) to the diagonal to guarantee positive definiteness. Condition numbers of the regularized matrices are reported in Table 1. Note that Σ is estimated separately for each library, so *d*_M_ values are comparable within a library but not across libraries. We then calculate the Mahalanobis distances *d*_M_ and Mahalanobis angles *θ*_M_ with Equations (3) and (5), respectively.
  2. **Tanimoto indices**. We quantify pairwise similarity to the target molecule with the TSI [Equation (7)].
5. **Visual inspection**. We prepare three complementary plots: (i) TSI versus *d*_M_, (ii) TSI versus *θ*_M_, and (iii) a polar *d*_M_ versus *θ*_M_ map to expose concordances and discrepancies between the discrete and continuous similarity measures, and we overlay LOWESS‐smoothed trend lines to guide interpretation.

### 3.2 Aspirin Case Study

We use a custom library of 31 393 aspirinoid, curcuminoid, or ibuprofenoid molecules (the ACI dataset) obtained from PubChem through substructure searches centered on the three parent cores [29]. We employed aspirin's Mol2Vec embedding as the reference vector and computed the corresponding TSI, *d*_M_, and *θ*_M_ values for each compound. We ranked the molecules according to each metric and conducted a qualitative analysis of chemotypes identified by the Mahalanobis criteria.

### 3.3 Aniline Case Study

We used the QM9 database [30] for the second benchmark of our MSI method. QM9 encompasses 133 885 small organic molecules comprising C, H, N, O, and F atoms and gives a wealth of information such as atomic charges and thermodynamic and electronic properties.

We applied a computational workflow identical to aspirin's to generate ECFP4 fingerprints and 300‐dimensional Mol2Vec embeddings for each molecular entry, designating aniline (benzenamine) as the reference compound. To address the substantially greater size of QM9 relative to the ACI dataset, we implemented parallel processing of similarity calculations and covariance‐matrix estimation through joblib to optimize computational performance. The generated scatterplots and polar maps demonstrate concordance with those obtained for aspirin, thus establishing independent cross‐dataset validation of the Mol2Vec–Mahalanobis methodology.

### 3.4 Curcumin and Ibuprofen Case Studies

We also performed a similarity search for curcuminoid and ibuprofenoid molecules using the aforementioned ACI dataset and the same workflow as in the aspirin case study.

### 3.5 Digitoxin Case Study

Our final case study used digitoxin as the reference molecule. Digitoxin‐type molecules retrieved from PubChem were appended to the ACI dataset and the combined set was refiltered, yielding 32 903 molecules (an increase of 1510 molecules once all stereoisomers within the digitoxin set were removed). The same similarity‐search methodology was applied.

## 4 Results and Discussion

### 4.1 Molecular Test Systems

**FIGURE 1:**
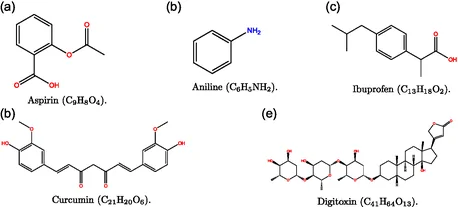
Reference molecules for MSI validation. Aspirin is a prototype NSAID featuring *ortho*‐substituted carboxylic acid and acetyl ester groups on a benzene ring; aniline is an aromatic amine with a primary amino group; curcumin is a polyphenolic natural product built from two *trans*‐cinnamoyl units flanking a 1,3‐diketone bridge; ibuprofen is a propionic‐acid NSAID; and digitoxin is a cardiac glycoside with a steroidal aglycone linked to a trisaccharide chain. These five structurally distinct molecules served as query compounds for evaluating MSI performance across pharmaceutical, industrial, natural‐product, and cardiac‐glycoside chemical spaces.

MSI was evaluated on five reference compounds chosen to span a wide range of size, functionality, and application domain. Aspirin ($C_{9}H_{8}O_{4}$, *M*_W_ = 180.16 Da), a salicylate NSAID with extensive clinical data, and ibuprofen ($C_{13}H_{18}O_{2}$, *M*_W_ = 206.28 Da), a propionic‐acid NSAID, represent small pharmaceuticals; curcumin ($C_{21}H_{20}O_{6}$, *M*_W_ = 368.38 Da) is a mid‐sized polyphenolic natural product; digitoxin ($C_{41}H_{64}O_{13}$, *M*_W_ = 764.94 Da) is a large, highly glycosylated cardiac glycoside; and aniline ($C_{6}H_{5}{\mathrm{NH}}_{2}$, *M*_W_ = 93.13 Da), an industrial aromatic amine with distinct electronic and optical properties, provides a small nonpharmaceutical test case for which independent quantum‐chemical reference data are available. Aspirin and aniline are analyzed in full in Sections 4.2 and 4.3; the three structurally more complex compounds are treated in Section 4.4. Figure 1 shows the molecular structure of each system.

### 4.2 Aspirin Case Study

Aspirin's therapeutic importance stems from COX enzyme inhibition, yielding analgesic, antipyretic, and anti‐inflammatory effects [45]. Beyond its traditional applications, low‐dose aspirin also serves as a cornerstone of cardiovascular prophylaxis [46]. To identify structurally related compounds, we screened the ACI library using both MSI and TSI metrics. This process revealed benzoic‐acid derivatives, which we label **BZ** ***n*** (***n *** = 1, …, 17), as all molecules identified as aspirin‐like by either metric shared this structural feature. Full IUPAC names for **BZ1**–**BZ17** are provided in the Supporting Information (molecules_names.pdf).

#### 4.2.1 MSI‐Based Ranking

In Mahalanobis space, equal‐*d*_M_ contours form ellipsoids defined by embedding covariance. The distance *d*_M_ quantifies statistical deviation after normalizing for correlated dimensions, while the angular term *θ*_M_ captures directional alignment in feature space.

**FIGURE 2:**
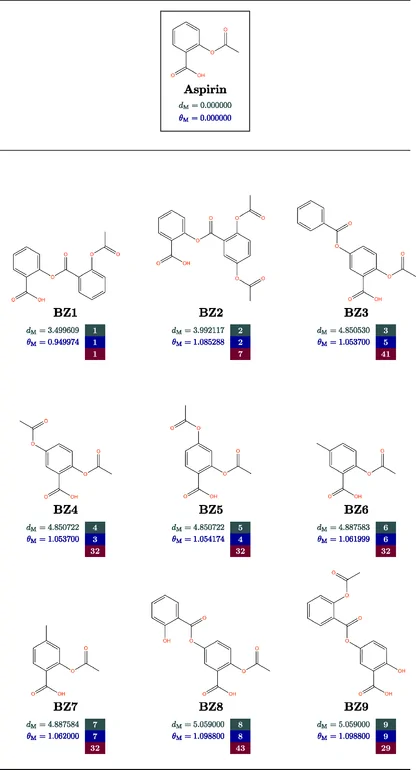
Top aspirin‐like molecules under a Mahalanobis metric in Mol2Vec space. At the top, we show the aspirin molecule as the reference compound. Panels labeled **BZ1**–**BZ9** depict the nine closest molecules. Each panel reports the Mahalanobis distance *d*_M_ (green) and the polar angle *θ*_M_ (blue) of the neighbor with respect to the aspirin vector in the metric space. The small three‐color badge summarizes ranks for each molecule (top to bottom: rank by *d*_M_, rank by *θ*_M_, and rank by TSI).

Figure 2 presents the nine most similar aspirin analogs ranked by MSI. Numerically, these neighbors span *d*_M _= 3.50–5.06 and *θ*_M _= 0.95°–1.10°. The closest analog (**BZ1**) lies at *d*_M _= 3.50, *θ*_M _= 0.95°; the remaining eight cluster tightly in angle (1.05°–1.10°), consistent with recognition of a common salicylate chemotype, while *d*_M_ increases stepwise from 3.99 to 5.06, reflecting successive functional modifications. All top‐ranked molecules preserve: (i) the *ortho*‐phenyl‐hydroxy‐(acyl/ester) motif, (ii) conjugated aryl systems, and (iii) hydrogen‐bonding capable groups.

The narrow spread of *θ*_M_ across the top‐ranked analogs confirms that their proximity to aspirin arises from consistent alignment in whitened space rather than from compensating displacements along opposite directions. Consistent with Equation (6), *d*_M_ and *θ*_M_ order these nine molecules almost identically; the angular coordinate is reported here because its bounded scale makes the thresholds of Section 4.6 portable between libraries, not because it separates molecules that *d*_M_ merges. The Mahalanobis metric accounts for feature covariance, downweighting redundant axes and emphasizing scaffold‐level differences.

#### 4.2.2 TSI‐Based Ranking

**FIGURE 3:**
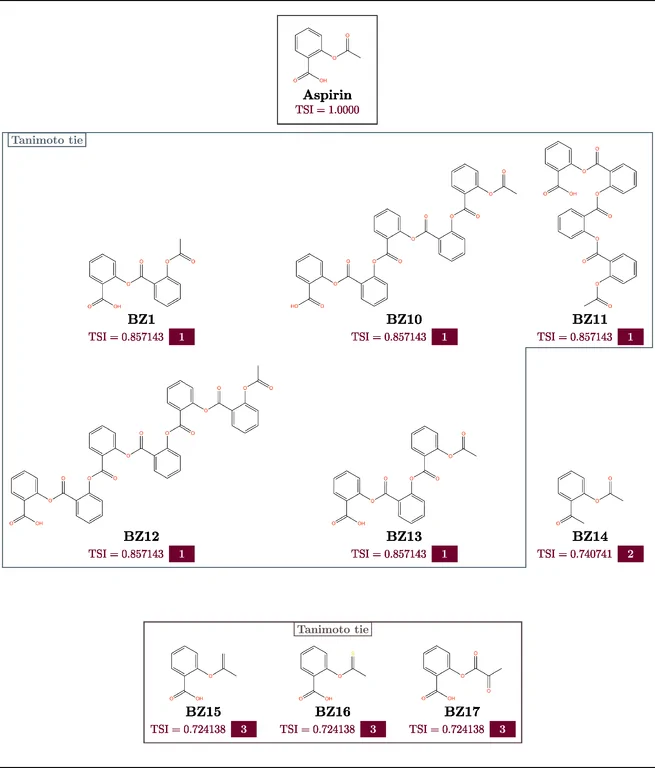
Top aspirin‐like molecules under Tanimoto similarity (TSI). The reference (aspirin) appears at the top with TSI = 1.0000. When candidates have the same Tanimoto similarity to aspirin, they are grouped into boxes labeled “Tanimoto tie”.

The nine MSI‐ranked molecules of Figure 2 carry seven distinct *d*_M_ values—**BZ4**/**BZ5** and **BZ8**/**BZ9** coincide to six decimal places—whereas the nine TSI‐ranked molecules of Figure 3 collapse onto only three distinct scores. If further significant figures are considered, Mahalanobis is able to distinguish between scores, whereas Tanimoto's stay tied. Rank 1 contains five molecules at TSI = 0.8571 (**BZ1**, **BZ10**, **BZ11**, **BZ12**, and **BZ13**), rank 2 a single molecule at TSI = 0.7407 (**BZ14**), and rank 3 a three‐way tie at TSI = 0.7241 (**BZ15**, **BZ16**, and **BZ17**).

The composition of the first plateau is diagnostic. **BZ1**, **BZ10**, **BZ11**, **BZ12**, and **BZ13** are salicylate oligomers—the dimer, trimer, and tetramer of the same acetylsalicyloyl unit—and they receive an identical Tanimoto score because ECFP4 records the *presence* of each circular substructure but not the number of times it occurs. Adding another repeat unit to an oligomer creates no new substructure environment at radius 2, so the bit set does not change and *the coefficient cannot change either*. This is a property of the binary representation rather than of the coefficient, and the point can be checked directly: the acetylsalicyloyl dimer, trimer, and tetramer each return TSI = 0.857143 against aspirin, and TSI = 1.000000 against *one another*, whereas the corresponding count‐based Morgan fingerprint (ECFC4, same radius and fold length) returns 0.5738 for the aspirin/dimer pair and 0.7011 for the dimer/trimer pair. The multiplicity‐weighted sum of Equation (2) behaves in the same way, and *d*_M_ separates all five molecules. Two further mechanisms contribute to the remaining plateaus: bit collisions introduced by folding the hashed fingerprint to 2048 bits, and the limited sensitivity of radius‐2 environments to regiochemical rearrangement. The same oligomer effect reappears, in a chemically very different setting, in the digitoxin case study of Section 4.4.3.

While TSI robustly retrieves the salicylate pharmacophore, extensive ties limit fine‐grained ordering necessary for lead selection or matched molecular series construction. TSI excels at broad neighborhood discovery but lacks the discriminatory power provided by continuous embedding‐based metrics.

#### 4.2.3 Metric Relationships

**FIGURE 4:**
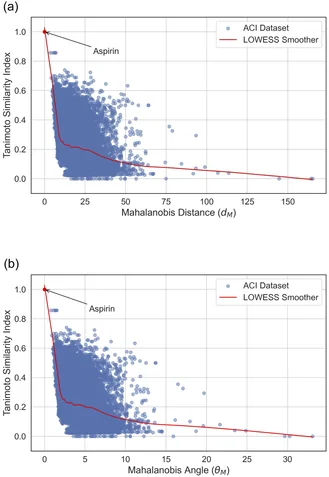
Relationship between fingerprint‐based Tanimoto similarity and two Mahalanobis‐metric descriptors for the ACI dataset using aspirin as the query molecule. (a) TSI versus Mahalanobis distance *d*_M_ in Mol2Vec space. (b) TSI versus Mahalanobis angle *θ*_M_ (in degrees). Blue points correspond to molecules in the ACI dataset; the red curve is a LOWESS smoother. Aspirin, compared to itself, is highlighted at TSI = 1, and *d*_M _= 0 and *θ*_M _= 0 for each panel, respectively. Both panels exhibit a strong, nonlinear inverse trend, characterized by an initial steep decay followed by a shallow tail. Grid lines aid visual comparison.

Figure 4 displays empirical correlations between TSI and Mahalanobis metrics. Both panels exhibit strong nonlinear inverse trends with two distinct regimes:

1. **High‐similarity zone** (*d*_M _≤ 20, *θ*_M _≤ 5°): Steep slopes mean that small Mahalanobis changes result in substantial TSI decreases, facilitating identification of close analogs. As either Mahalanobis value increases, the transition toward the next regime is marked by the slope's gradual flattening.
2. **Low‐similarity basin** (beyond the inflection point): In this regime, TSI approaches zero, whereas Mahalanobis metrics maintain discriminatory power by capturing higher order correlations between molecular descriptors that are not detected by fingerprint‐based methods (which rely on binary structural similarity).

TSI's propensity to similarity ties is visible in Figure 4: the earliest iso‐TSI slice occurs at TSI = 0.857 with molecules **BZ1** and **BZ10** to **BZ13**, whereas both Mahalanobis indices rank these five molecules differently. Outliers with moderate TSI but extreme Mahalanobis values reveal that fingerprint‐based (TSI) and embedding‐driven (*d*_M_, *θ*_M_) metrics assess distinct, orthogonal aspects of molecular similarity.

**FIGURE 5:**
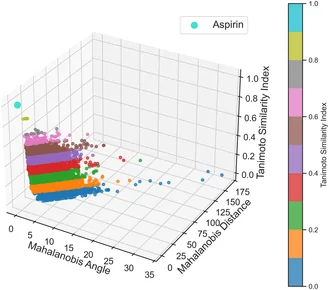
Aspirin‐referenced 3D map of similarity. TSI (vertical axis and color scale) as a joint function of Mahalanobis angle *θ*_M_ (*x*‐axis, degrees) and Mahalanobis distance *d*_M_ (*y*‐axis) in Mol2Vec space for the ACI library. The cyan marker highlights aspirin (self‐comparison) at TSI = 1 and *d*_M _= *θ*_M _= 0. Horizontal color bands correspond to iso‐TSI “slices,” revealing how TSI decays with both *d*_M_ and *θ*_M_.

The 3D representation (Figure 5) integrates these insights: high TSI requires both small *d*_M_ and small *θ*_M_. Iso‐TSI slices visualize analog collapse to identical scores, which embedding metrics resolve through continuous variation. The steep high‐TSI ridge at low separations defines a natural retrieval zone; beyond this, additional separation negligibly affects already‐low TSI.

Figure 5 illustrates metric relationships in three dimensions. Most points fall at small angles (≲10°) and short distances (≲30); in contrast, large *d*_M_ or *θ*_M_ values are associated with low TSI near zero. High fingerprint similarity requires joint constraints: molecules must be both close (small *d*_M_) and directionally aligned (small *θ*_M_). The layered structure—iso‐TSI slices—exposes the Tanimoto tie problem, where chemically distinct molecules receive identical scores. Embedding‐based metrics resolve these ties through continuous distance and angle values. The high‐TSI ridge at low Mahalanobis separations marks a natural analog retrieval zone, beyond which TSI flattens to near‐zero regardless of further separation.

**FIGURE 6:**
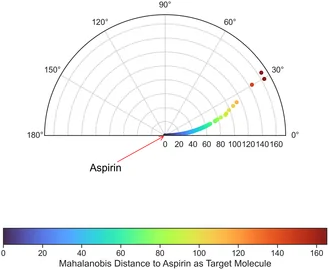
Polar representation of molecular embeddings around aspirin. The radial coordinate reports the Mahalanobis distance (*d*_M_) to aspirin in the Mol2Vec embedding space (0–160). The angular coordinate (*θ*; 0°–180°) reports the Mahalanobis angle, which quantifies the directional deviation of a molecule's embedding from aspirin's vector direction in the same metric space. Point color redundantly encodes *d*_M_ to ease visual parsing.

The polar plot (Figure 6) reveals that 90% of the ACI library lies within the region bounded by *θ*_M _< 10° and *d*_M _< 80. The narrow fan‐shaped distribution, with a maximum angle *θ*_M _≈ 30° and maximum distance *d*_M _≈ 160, and with no molecules beyond *θ*_M _= 90°, confirms all candidates align with aspirin in embedding space. Applying joint thresholds efficiently filters analogs: restricting angle *θ*_M_ to ≤5° confines the distance *d*_M_ to ≤20, yielding high‐priority similar molecules. Two outliers at *θ*_M _> 30° and *d*_M _> 140 occupy a sparsely populated region of the embedding space and are, on that basis alone, candidate starting points for scaffold hopping, despite their low Tanimoto scores. We note that this is a hypothesis generated by the geometry, not a validated result: no biological or physicochemical assessment of these two compounds is presented here.

### 4.3 Aniline Case Study

Aniline serves as a crucial precursor for synthetic dyes, pharmaceuticals, and polyurethane polymers [47]. Its UV–vis absorption around 280 nm and fluorescence properties make it valuable for photochemical applications [48, 49]. Polyaniline derivatives show promise for OLEDs, OPVs, and sensors due to tunable conductivity and optical properties [50, 51, 52].

Following the aspirin methodology, we applied both MSI and TSI to identify aniline analogs within the QM9 dataset. The top 15 structurally similar compounds, labeled **NH*n*** (***n *** = 1, …, 15), span several chemical classes: simple aromatics (**NH1**, **NH4**, **NH5**, **NH7**), toluidines (**NH2**, **NH9**), phenylenediamines (**NH3**, **NH8**, **NH10**), aminophenols (**NH6**, **NH14**), aminocycloheptatrienone isomers (**NH11**–**NH13**), and a halogenated aniline (**NH15**). Compound names for **NH1**–**NH15** are listed in the Supporting Information (molecules_names.pdf).

Furthermore, the QM9 dataset lists the HOMO–LUMO gap (*E*_gap_) of every molecule, so we consider the difference between the gap of each candidate and that of aniline, denoted Δ*E*_gap_:



(9)

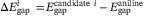




so that a positive Δ*E*_gap_ denotes a candidate whose frontier‐orbital gap is *wider* than aniline's, and a negative value a narrower (bathochromically shifted) gap. This is the convention used in all figures and tables of this work.

#### 4.3.1 MSI Versus TSI Performance

**FIGURE 7:**
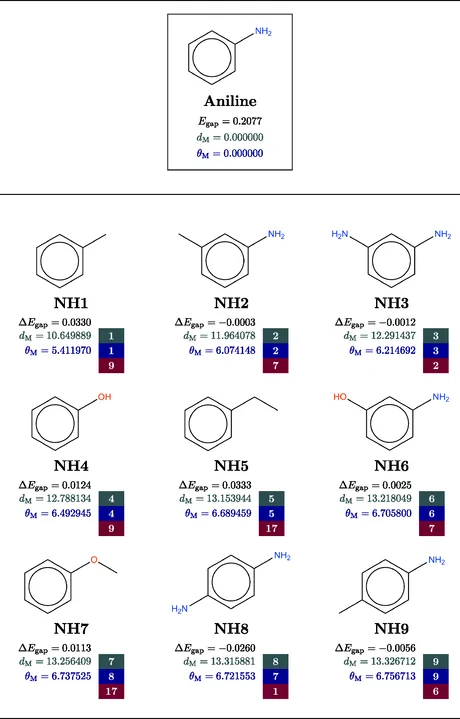
Top anilinoid molecules under MSI. Aniline (top panel) serves as reference with *E*_gap _= 0.2077*E*_h_), *d*_M _= 0.0000, and *θ*_M _= 0.0000°. Each of the nine analogs (**NH1**–**NH9**) reports its structure, difference in HOMO–LUMO gap compared to aniline (Δ*E*_gap_), Mahalanobis distance (*d*_M_), and Mahalanobis angle (*θ*_M_). Color‐coded rectangular badges show three ranking systems: Mahalanobis distance (green), Mahalanobis angle (blue), and TSI (red).

Figure 7 reveals systematic ranking discrepancies. Toluene (**NH1**) achieves *d*_M_ rank 1 but only TSI rank 9 since it lacks the amino group on which the fingerprint overlap largely depends. The widest divergences are those of ethylbenzene (**NH5**, *d*_M_ rank 5 vs. TSI rank 17) and anisole (**NH7**, *d*_M_ rank 7 vs. TSI rank 17), both of which the fingerprint places far outside its top ten. Conversely, *p*‐phenylenediamine (**NH8**) ranks 1st by TSI but 8th by *d*_M_, reflecting the sensitivity of the fingerprint to substructural overlap even though **NH8** shows a substantial electronic perturbation (Δ*E*_gap _= −0.0260*E*_h_).

Mahalanobis clustering patterns emerge across chemical families. For example, methylated anilines (**NH2**, **NH9**) cluster at *d*_M _= 11.96–13.3 and *θ*_M _= 6.0°–6.8°, with near‐zero Δ*E*_gap_ values. In contrast, the oxygen‐substituted analogs phenol (**NH4**) and anisole (**NH7**) occupy a distinct region at *d*_M _= 12.8–13.3 and *θ*_M _= 6.5°–6.7°. Both show positive Δ*E*_gap_ shifts (+0.0113*E*_h_ to +0.0124*E*_h_), that is, a widening of the gap relative to aniline. This is the expected direction: hydroxy and methoxy substituents are weaker π‐donors than the amino group and possess greater *σ*‐electron‐withdrawing character, so replacing NH_2_ by OH or OCH3 stabilizes the HOMO and widens the frontier‐orbital gap. 3‐Aminophenol (**NH6**), which retains the amino group alongside a hydroxyl, shows a much smaller shift (+0.0025*E*_h_), as expected on the same reasoning. That MSI rankings vary systematically with the HOMO–LUMO gap is notable because the Mol2Vec model was trained on structural context alone, with no exposure to quantum‐chemical data; the correspondence is therefore an emergent property of the embedding rather than something it was fitted to reproduce. This electronic–structural relationship deserves further exploration.

**FIGURE 8:**
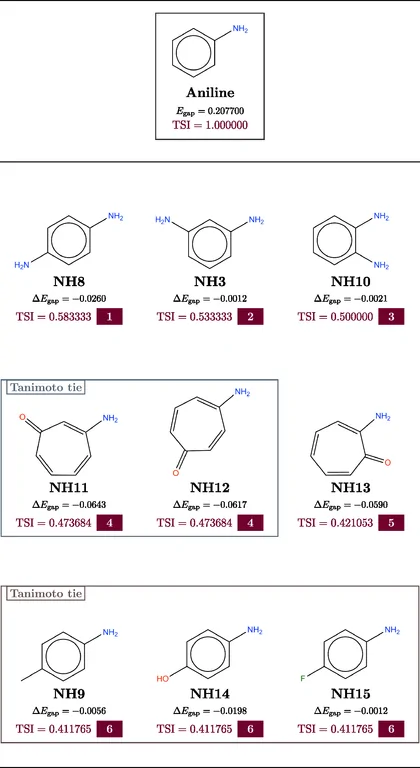
Top‐ranked aniline analogs from the QM9 database using Tanimoto Similarity Index (TSI). Aniline (top) serves as a reference with *E*_gap _= 0.2077*E*_h_ and TSI = 1.0000. Nine highest‐ranked analogs are shown with TSI scores (red rectangular badges), including phenylenediamines (**NH8**, **NH3**, **NH10**), aminocycloheptatrienone (aminotropone) isomers (**NH11**–**NH13**), and monosubstituted anilines (**NH9**, **NH14**, **NH15**). Square boxes highlight Tanimoto tie cases where multiple molecules receive identical TSI scores despite differing structures and electronic properties.

Figure 8 displays TSI‐ranked analogs. Disubstituted phenylenediamines (**NH8**, **NH3**, **NH10**) achieve highest scores (TSI = 0.50 to 0.58) through maximum substructural overlap. Critical limitations arise from Tanimoto ties: **NH11** and **NH12** both score TSI = 0.4737 despite different substitution patterns and electronic properties. More severe ties at rank 6 assign identical TSI = 0.4118 to **NH9** (methyl, Δ*E*_gap _= −0.0056*E*_h_), **NH14** (hydroxyl, Δ*E*_gap _= −0.0198*E*_h_), and **NH15** (fluorine, Δ*E*_gap _= −0.0012*E*_h_).

The aminotropones **NH11**–**NH13** show the largest gap reductions (Δ*E*_gap _= −0.064*E*_h_ to −0.059*E*_h_) yet receive only moderate Tanimoto scores (0.42–0.47), so that in this dataset substructural overlap is a poor predictor of frontier‐orbital similarity. A reduction of the HOMO–LUMO gap corresponds to a bathochromic (red) shift, that is, a displacement of the lowest absorption band toward longer wavelengths, and here it reflects the extended, nonbenzenoid π system of the tropone ring.

TSI elevates molecules based on functional group count rather than physicochemical relevance. Comparison with MSI reveals orthogonal information: toluene and ethylbenzene rank highly by MSI due to aromatic electronic environment matching, but are absent from TSI top‐9.

**FIGURE 9:**
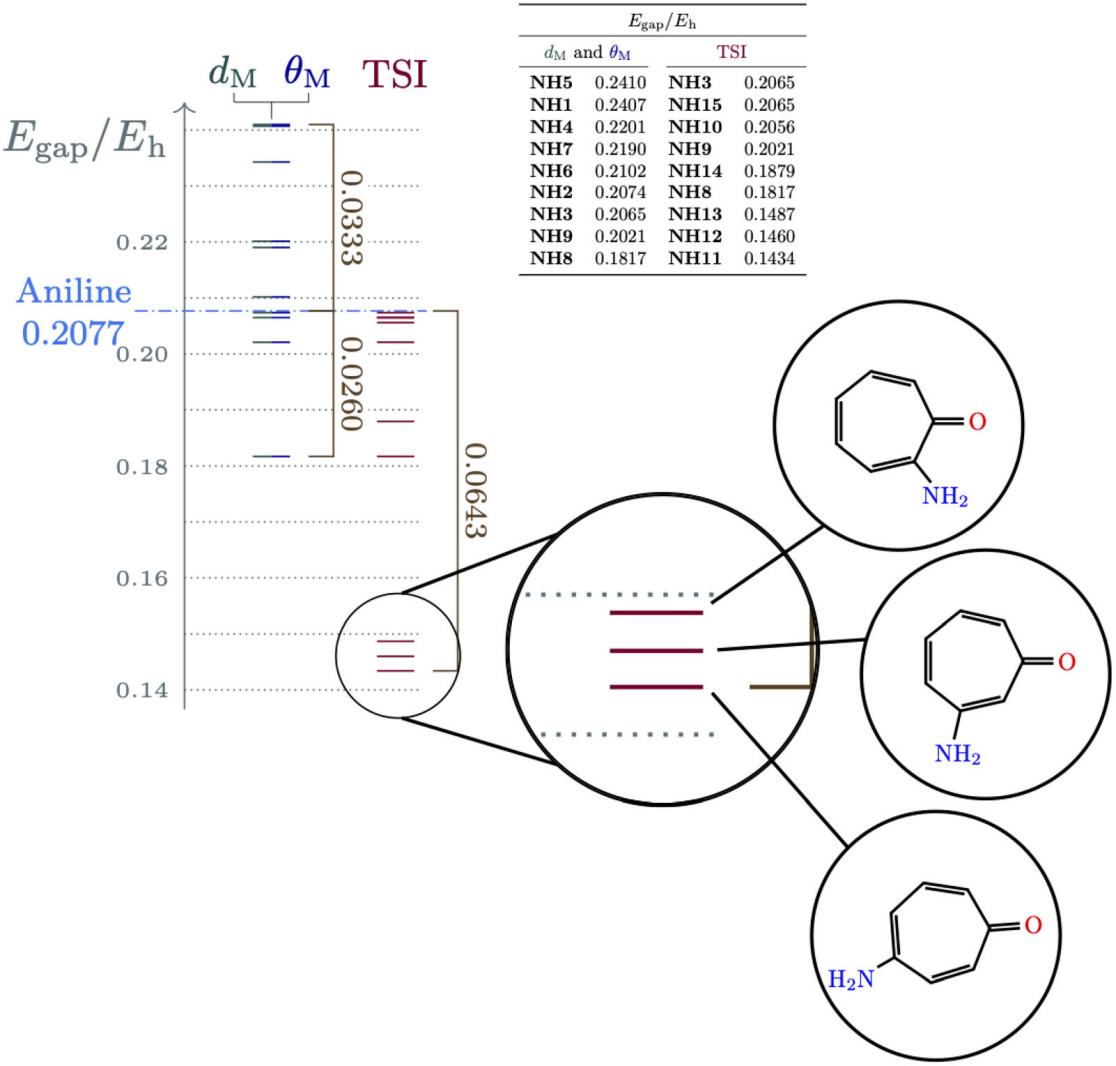
Ranking discrepancies between Mahalanobis‐based and fingerprint‐based similarity metrics for aniline analogs. Left panel displays HOMO–LUMO gap in Hartree (*E*_gap_/*E*_h_) distributions for top‐ranked molecules using Mahalanobis distance and angle (*d*_M_ and *θ*_M_, blue bars) versus Tanimoto Similarity Index (TSI, red bars). Aniline reference is marked at 0.2077*E*_h_ (blue dashed line). Right table lists molecules ranked by combined Mahalanobis metrics versus TSI. Three aminocycloheptatrienone isomers are described in the magnified circles.

Figure 9 compares MSI and TSI rankings for aniline analogs, taking the *E*_gap_ of this molecule as the reference. Mahalanobis‐ranked molecules (blue) cover an *E*_gap_ range of 0.1817*E*_h_–0.2410*E*_h_. TSI‐ranked molecules (red) cluster within 0.1434*E*_h_–0.2065*E*_h_, mainly below aniline's reference value (0.2077*E*_h_). Only three molecules (**NH3**, **NH8**, **NH9**) appear in both nine‐molecule lists, indicating a systematic difference between the two ranking methods.

The three aminocycloheptatrienone (aminotropone) isomers **NH11**–**NH13** illustrate the consequences of this difference. They occupy TSI ranks 4–5, two of them sharing an identical score, yet their electronic structures are appreciably different from aniline's and from each other (Δ*E*_gap _= −0.0643*E*_h_, −0.0617*E*_h_, and −0.0590*E*_h_, the three largest gap reductions in the entire candidate set). The fingerprint promotes them because a seven‐membered aminoketone reproduces many of the same radius‐2 environments as an aminobenzene, while MSI places all three outside its top 10: in the embedding space, the nonbenzenoid tropone ring is a genuinely different structural context and the vectors are correspondingly displaced. Which behavior is preferable depends on the objective. Where *electronic traits* rather than *substructural overlap* determine function—optoelectronic screening, or the search for compounds of comparable reactivity—the MSI ordering is the more useful one. We note, however, that MSI does distinguish between these three isomers, and assigns them the scores {14.731708, 15.719437, 14.521427}.

#### 4.3.2 QM9 Chemical Space Analysis

**FIGURE 10:**
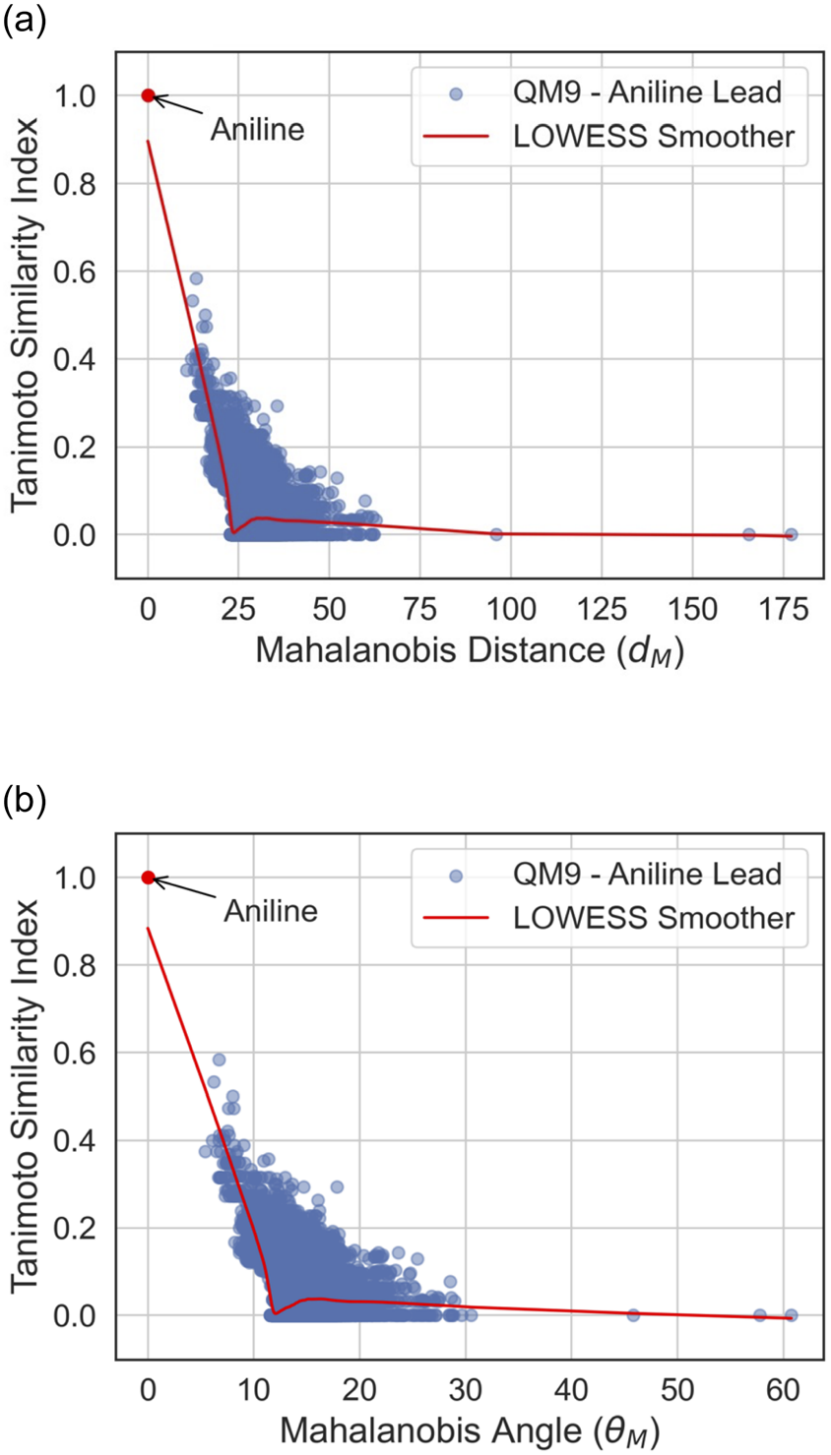
Scatter plots display the relationship between fingerprint‐based Tanimoto similarity and two Mahalanobis‐metric descriptors—distance and angle—in the QM9 dataset, with aniline as the target molecule. Panel (a) presents Tanimoto similarity versus Mahalanobis distance (*d*_M_). Panel (b) shows Tanimoto similarity versus Mahalanobis angle (*θ*_M_). Blue markers represent QM9 molecules, while the red‐filled circle marks the target. The red line in each panel is a LOWESS fit, highlighting the trend. Grid lines aid visual comparison.

Figure 10 shows TSI versus Mahalanobis metrics for QM9. The LOWESS curves exhibit sharp decay within *d*_M _≲ 20 or *θ*_M _≲ 10°, showing a strong inverse correlation. Beyond *d*_M _≈ 25 or *θ*_M _≈ 15°, TSI values remain close to zero for further increases in Mahalanobis distance or angle, indicating diminished sensitivity of TSI. However, Mahalanobis metrics continue to separate compounds beyond this range as their values increase further. This defines an applicability domain: compounds within the Mahalanobis envelope retain moderate‐to‐high TSI and are promising analogs. The angular metric *θ*_M_ provides similar discrimination on a compressed scale, helpful in high‐dimensional spaces where high dimensionality inflates *d*_M_.

**FIGURE 11:**
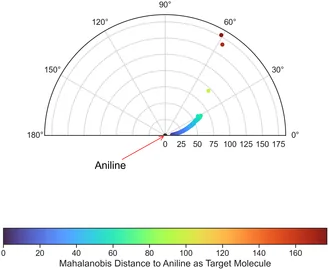
Polar representation of Mahalanobis geometry for the QM9 dataset using aniline as reference. Each point represents an individual molecule. The azimuthal coordinate represents the Mahalanobis angle *θ*_M_ in degrees, while the radial coordinate shows the Mahalanobis distance *d*_M_. Marker color shows the corresponding *d*_M_ value, as indicated in the color bar to the right. The query molecule, aniline, is positioned at the origin and highlighted with a red arrow. Gray concentric circles, spaced every 25 distance units, and radial grid lines every 30° support visual inspection.

The polar plot (Figure 11) shows that 40% of QM9 data points are concentrated within *d*_M_ < 25 and *θ*_M _< 10°, reflecting substantial alignment with aniline. The intermediate region, defined by 25 ≤ *d*_M _≤ 75 and 10° ≤ *θ*_M _≤ 25°, forms a smooth arc. Here, the slope of approximately 0.25° per distance unit means that for each unit increase in *d*_M_, *θ*_M_ increases by about 0.25°, indicating that dissimilarity increases mainly through descriptor reorientation. A few outliers with *d*_M _> 100 and *θ*_M _≈ 60° occupy the outer regions of chemical space.

Beyond *d*_M _∼ 75, the distribution becomes steeper and splits into separate angular groups, showing anisotropy in feature space: leaving the main chemical path allows for several distinct directions. Dual thresholds (*d*_M _≤ 50, *θ*_M _≤ 15°) identify molecules with similar direction and preserved pharmacophoric motifs, while the sparse region at *θ*_M _≥ 45° and *d*_M _≥ 100 acts as a source for scaffold‐hopping.

**FIGURE 12:**
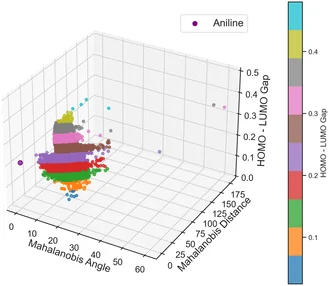
Three‐dimensional correlation between Mahalanobis geometry and frontier‐orbital energetics for the QM9 dataset centered on aniline. Cartesian axes display the Mahalanobis angle (*θ*_M_, abscissa), the Mahalanobis distance (*d*_M_, ordinate), and the HOMO–LUMO gap (*E*_gap_, vertical). Points are color‐coded to correspond to their *E*_gap_ values as shown in the side bar. The reference molecule (aniline) is highlighted in purple at the coordinate origin.

Figure 12 correlates Mahalanobis geometry with HOMO–LUMO gaps. The cone‐like distribution reveals three trends: (i) coupled radial‐angular expansion with most points below *θ*_M_ = 25° and *d*_M _= 75, (ii) electronic stratification in discrete terraces with steps of ∼0.05*E*_h_–0.07*E*_h_, and (iii) sparse outliers at *d*_M _> 125 and *θ*_M _≥ 40° with *E*_gap _≳ 0.30*E*_h_.

Within the inner neighborhood, where Mahalanobis distance *d*_M_ is less than 25 and Mahalanobis angle *θ*_M_ is less than 10°, the gap varies minimally. This means the compounds are very similar by this metric. Local analogs—structurally similar molecules—preserve electronic profiles. In contrast, outside this region, both the Mahalanobis distance and angle increase, leading to broader electronic gaps (energy differences between molecular orbitals). This signals a shift in electronic similarity. These results validate the use of Mahalanobis geometry—a method for quantifying how unusual a molecule is compared to a reference group—to predict electronic consequences. Compounds with low angle and distance retain comparable chemical reactivity, while those with higher values show altered electronic properties. Stepwise gap changes reflect discrete substitutions of functional groups (distinct sets of atoms defining a molecule's reactivity) in the QM9 molecular dataset. High‐gap outliers identify molecules suitable for scaffold hopping, a process that discovers new chemotypes by identifying novel molecular core structures. Overall, this aids in searching for electronically distinct core structures (chemotypes).

An important limitation shared by all similarity‐based approaches, including MSI, is the existence of *activity cliffs*: pairs or groups of structurally similar compounds that display large differences in biological activity [53]. Activity cliffs represent well‐known exceptions to the Similar Property Principle and arise from highly localized structure–activity relationships where small chemical modifications produce disproportionate changes in potency. While MSI's continuous scoring provides finer discrimination than TSI and can better resolve near‐neighbor relationships, it does not inherently predict whether a structural perturbation will generate an activity cliff. A systematic investigation of activity cliff distributions within MSI's dual radial‐angular applicability domain represents a valuable direction for future work.

Three advantages of MSI over the ECFP4 + Tanimoto baseline emerge from these two case studies. First, it promotes chemically reasonable analogs that the fingerprint deprioritises—toluene ranks 1st by *d*_M_ but 9th by TSI for aniline. Second, it resolves ranking ties, because the embedding varies continuously where the bit set does not. Third, its geometry varies systematically with the HOMO–LUMO gap, even though the embedding never saw electronic data.

The Mahalanobis coordinates also delimit a practical applicability domain (*d*_M _≲ 20–25, *θ*_M _≲ 5°–10°) for high‐similarity retrieval, and retain discriminatory power in the regime where the Tanimoto coefficient has saturated near zero. Polar and three‐dimensional visualizations make these thresholds easy to set and reveal the anisotropy of the sampled space.

We are careful not to overstate what these two case studies establish. They show that the Mahalanobis geometry of a Mol2Vec embedding varies with electronic structure in a chemically interpretable way; they do not demonstrate predictive accuracy for any specific property, and no regression model or held‐out test is presented. The extension to three structurally more complex reference compounds in Section 4.4 addresses the breadth of the evaluation; the question of predictive validity is left to future work (Section 4.6).While the present study emphasizes pharmaceutical and optoelectronic applications, molecular similarity has a far broader scope. MSI is equally applicable to materials science (e.g., organic semiconductor design and polymer informatics), toxicology (structure–activity modeling of toxic endpoints), agrochemistry, and chemical biology. The data‐driven character of Mol2Vec embeddings ensures generalizability across chemical domains without retraining the embedding model, provided that the training corpus adequately represents the target chemical space of interest.

### 4.4 Extended Validation: Structurally Complex Reference Molecules

Two reference compounds are a narrow basis on which to claim general applicability, and we therefore repeated the analysis of Section 3 on three additional molecules chosen to be larger and structurally more complex, keeping the pipeline unchanged throughout (Mol2Vec embedding, covariance estimation, *d*_M_, *θ*_M_, and ECFP4 + Tanimoto). Two of them, **curcumin** (C21H20O6, *M*_W_ = 368.4 Da, a polyphenolic natural product with two *trans*‐cinnamoyl units flanking a 1,3‐diketone bridge) and **ibuprofen** (C13H18O2, *M*_W_ = 206.28 Da, a propionic‐acid NSAID), were queried against the same 31 393‐molecule ACI library used for aspirin. Because curcumin and ibuprofen are themselves the C and I of that acronym, each also gives us a chance to check that the embedding space is internally consistent: querying on curcumin recovers aspirin and ibuprofen within *d*_M _< 11, and querying on ibuprofen recovers aspirin and curcumin within the same distance, so the three parent scaffolds sit in one connected, densely populated region rather than three separate islands. The third addition, **digitoxin** (C41H64O13, *M*_W_ = 764.94 Da), is a cardiac glycoside bearing no structural relationship to the other three: it belongs to none of the aspirin, curcumin, or ibuprofen families and is between two and four times their molecular weight. For this compound, we combined the existing ACI library with an additional set of digitoxin‐type molecules retrieved from PubChem, yielding a combined library of 32 903 molecules after removing invalid SMILES, entries with out‐of‐vocabulary Mol2Vec identifiers, and stereochemical duplicates (covariance condition number 8.37 × 10^3^).

#### 4.4.1 Curcumin as Query Molecule

Curcumin's dataset‐wide statistics sit close to aspirin's (Table 1): a log‐normal distribution again gives the best fit for TSI, *d*_M_, and *θ*_M_, *d*_M_ and *θ*_M_ are still nearly perfectly rank‐correlated (*ρ *= 1.0000), and TSI anticorrelates with the Mahalanobis descriptors to a similar degree as for aspirin (*r *= −0.2721 for TSI vs. *d*_M_).

**FIGURE 13:**
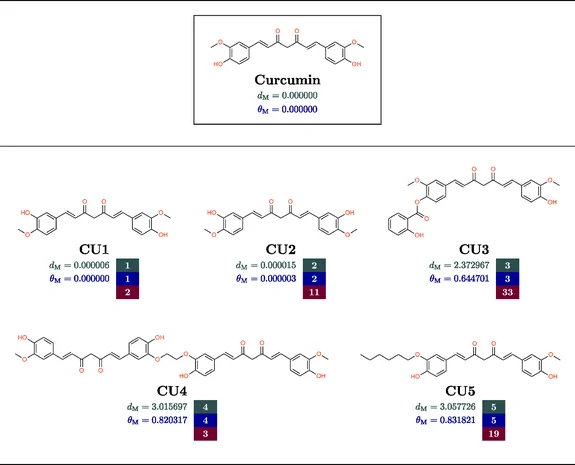
Five closest curcumin analogs ranked by MSI (Mahalanobis distance *d*_M_). Each panel reports rank, *d*_M_, *θ*_M_ (degrees), and an index badge for the corresponding neighbor in the ACI library. These are the 5 closest of the 10 molecules discussed in the text; compare with Figure 14, ranked by TSI on the same library.

**FIGURE 14:**
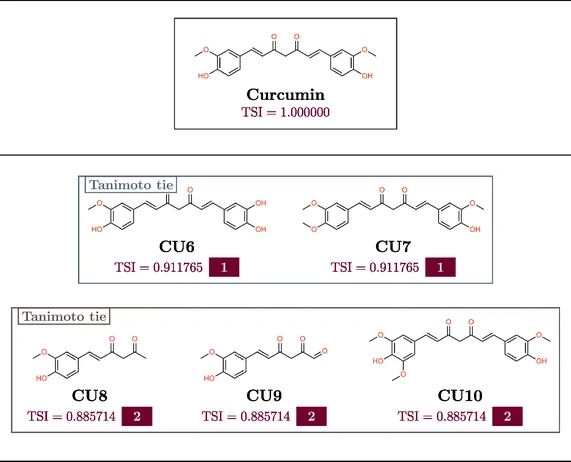
Five closest curcumin analogs ranked by the Tanimoto Similarity Index (TSI). Boxed groups mark Tanimoto ties: the first pair shares TSI = 0.9118, and the remaining three share TSI = 0.8857; across the full top‐10 comparison discussed in the text, seven molecules in total share this latter score, illustrating the ranking‐tie problem that MSI resolves (cf., Figure 13).

The tie‐breaking result carries over cleanly. Considering curcumin's 10 nearest neighbors, TSI collapses down to just 3 distinct scores (7 of the 10 molecules share TSI = 0.8857), while MSI still tells 9 of the 10 apart. Figures 13 and 14 show the five closest neighbors for each ranking. The two MSI‐ranked neighbors at *d*_M _≈ 0 (**CU1**, **CU2**) are near‐duplicate curcumin entries already present in the PubChem‐derived library, differing only in the placement of the methoxy and hydroxy substituents on the aromatic rings. MSI assigns both a distance near‐indistinguishable from zero, whereas the fingerprint separates them (TSI ranks 2 and 11). The direction of this disagreement should be read carefully: it is not a fingerprint failure but the opposite of the digitoxin case discussed in Section 4.4.3, and it shows that the Mol2Vec embedding used here is relatively insensitive to the positional isomerism of small substituents on an otherwise conserved scaffold. Where such regiochemistry matters, the fingerprint is the more discriminating representation, and the two should be used together.

#### 4.4.2 Ibuprofen as Query Molecule

Ibuprofen tells much the same story (Table 1): log‐normal is again the best‐fitting distribution for all three metrics, *d*_M_ and *θ*_M_ stay essentially colinear (*ρ *= 1.0000), and TSI anticorrelates with the Mahalanobis descriptors, a bit more weakly this time (*r *= −0.2176).

**FIGURE 15:**
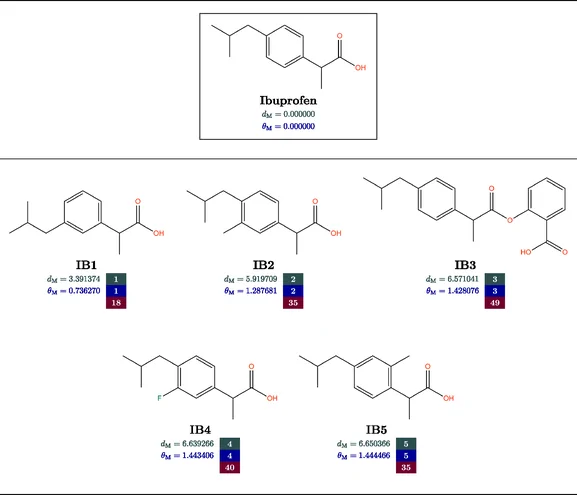
Five closest ibuprofen analogs ranked by MSI (Mahalanobis distance *d*_M_). Each panel reports rank, *d*_M_, *θ*_M_ (degrees), and an index badge, together with the molecule's ordinal Tanimoto rank across the full library (maroon badge). All five *d*_M_ values are distinct, in contrast with the TSI ranking of Figure 16.

**FIGURE 16:**
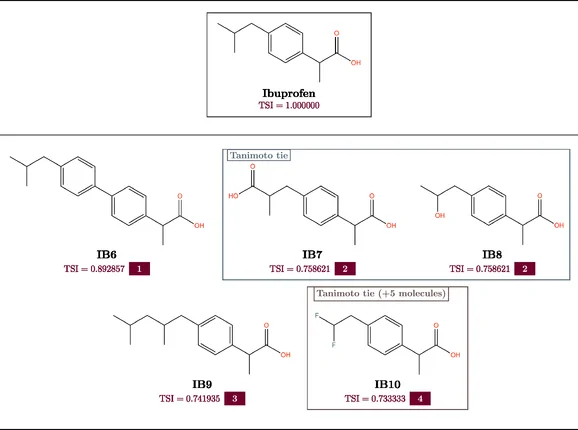
Five closest ibuprofen analogs ranked by TSI. A boxed pair already shares an identical score; the boxed group at rank 4 shares TSI = 0.7333 together with five additional molecules not pictured individually. In the full top‐10 comparison discussed in the text, 6 of the 10 molecules—mostly alkoxy‐chain homologs—share this score, the most severe tie‐collapse among the three ACI reference molecules studied.

If anything, the tie problem is worse here than for aspirin or curcumin. Across the 10 nearest neighbors, 6 of the top‐10 TSI hits share a single score, TSI = 0.7333, leaving only 4 distinct values; MSI, on the same 10 molecules, gives 10 distinct *d*_M_ values that increase steadily from 3.39 to 6.98. Figures 15 and 16 show the five closest neighbors by each metric; the TSI‐tied group is visible directly in Figure 16, where a boxed annotation marks the five additional molecules sharing that score but not pictured individually. Several of the tied molecules turn out to be simple chain‐length homologs, esters differing only in alkoxy tail length, and MSI orders them correctly by that increasing chain length while TSI cannot tell them apart at all.

#### 4.4.3 Digitoxin as Query Molecule

We augmented the ACI library with an additional set of digitoxin‐type molecules retrieved from PubChem. After removing all molecules with out‐of‐vocabulary Mol2Vec identifiers or stereochemical duplicates, the combined library comprised 32 903 molecules. Across this library, *d*_M_ ranges from 0 to 164.57 with a mean of 18.19 (variance 36.17), and TSI covers the full [0, 1] range, numbers broadly in line with the aspirin, curcumin, and ibuprofen datasets (Table 1).

**FIGURE 17:**
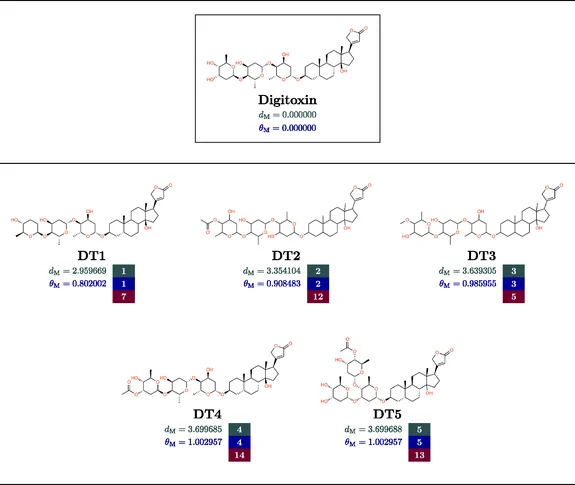
Five closest digitoxin analogs ranked by MSI (Mahalanobis distance *d*_M_). Each panel reports rank, *d*_M_, *θ*_M_ (degrees), and an index badge for the corresponding neighbor in the digitoxin‐focused library. **DT4** and **DT5** share an identical *d*_M _= 3.70, corresponding to regiochemical acetoxy‐position isomers of the same digitoxigenin‐trisaccharide skeleton (see text); a third such isomer falls just outside the five neighbors shown here.

Figure 17 shows digitoxin's five closest neighbors by MSI, drawn from the same ten‐molecule comparison used throughout this section. TSI again produces a tie‐heavy top‐10, with only 7 distinct scores out of 10 (a 3‐way tie at TSI = 0.9067, a 2‐way tie at TSI = 0.8889). This time, though, MSI is not completely tie‐free either: 3 of the 10 nearest neighbors by *d*_M_ land on exactly the same distance, 3.6997, 2 of which (**DT4** and **DT5**) appear among the 5 neighbors plotted in Figure 17, so MSI resolves 8 distinct values out of 10 rather than the 9–10 seen for curcumin and ibuprofen. All three share the same molecular weight (806.987 Da) and an almost identical digitoxigenin‐trisaccharide skeleton, differing only in which sugar ring carries an acetoxy group—a regiochemical difference that the 300‐dimensional Mol2Vec embedding apparently does not resolve for this scaffold family. MSI still resolves more distinct values than TSI on this query, 8/10 against 7/10, and treating three near‐identical regiochemical variants as equidistant is arguably the more defensible behavior since any ordering imposed on them would be arbitrary. The result nevertheless sets a clear bound on the method: the tie‐breaking advantage of MSI held in direction for all four ACI‐library reference compounds, but its magnitude is limited by what the underlying embedding can distinguish, and positional isomerism of small substituents on a conserved scaffold is precisely what this embedding distinguishes least well (compare the curcumin near‐duplicates of Section 4.4). This limitation is revisited in Section 4.6.

**FIGURE 18:**
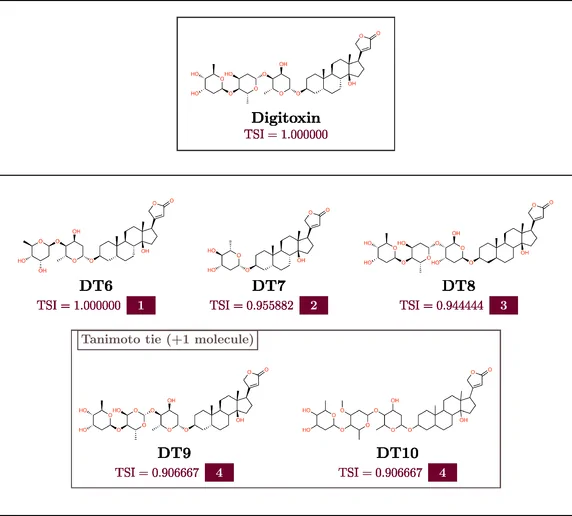
Five closest digitoxin analogs ranked by TSI. DT6, the top‐scoring match at TSI = 1.0000, is the *bis*‐digitoxoside homolog of digitoxin—one sugar unit shorter than the query—discussed in the text and shown alongside digitoxin in Figure 19. The boxed group at rank 4 (**DT9**, **DT10**) marks a Tanimoto tie at TSI = 0.9067 shared with one additional molecule not pictured individually.

**FIGURE 19:**
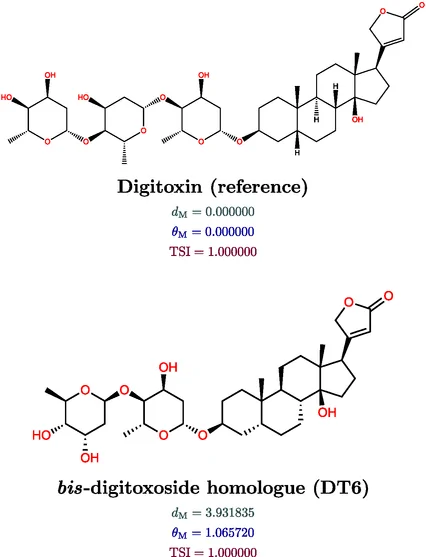
A Tanimoto‐indistinguishable homolog, resolved by MSI. Left: digitoxin, the query, whose digitoxigenin aglycone carries a chain of three digitoxose units. Right: **DT6**, the corresponding *bis*‐digitoxoside, carrying only two. Both molecules contain the same terminal and internal sugar environments, so every radius‐2 circular substructure present in one is present in the other and their ECFP4 bit sets are identical; the Tanimoto coefficient therefore returns TSI = 1.0000 for a molecule that differs from the query by an entire monosaccharide. Because the Mol2Vec molecule vector sums substructure embeddings with multiplicity (Equation (2) ), MSI separates them at *d*_M _= 3.93, *θ*_M _= 1.07°. This is the same failure mode seen for the salicylate oligomers of Figure 3.

Figure 18 shows the complementary TSI‐ranked neighbors, and the top‐scoring molecule is the most instructive result of this case study. **DT6** receives TSI = 1.0000, the maximum possible score, exactly as if it were the query itself. It is not: as Figure 19 makes clear, **DT6** is the *bis*‐digitoxoside homolog of digitoxin, carrying two digitoxose units on the digitoxigenin aglycone instead of three.

The reason the fingerprint cannot see the difference is the same one identified for the salicylate oligomers in Section 4.2.2. Digitoxin's sugar chain contains one terminal and two internal digitoxose rings; the *bis* homolog contains one terminal and one internal ring. Every circular substructure environment of radius 2 present in one molecule is therefore also present in the other—removing an internal repeat unit creates no new environment and eliminates none—so the two molecules generate identical ECFP4 bit sets—we verified that the folded 2048‐bit vectors agree in every position—and the Tanimoto coefficient is obliged to return unity. The coefficient is not at fault; a set‐based representation simply does not record how many times an environment occurs. Replacing the binary fingerprint by its count‐based counterpart while holding radius, fold length, and coefficient fixed lowers the score to 0.8428, which confirms that multiplicity, and not the choice of coefficient, is the operative variable.

MSI, in contrast, places **DT6** at *d*_M _= 3.93, *θ*_M _= 1.07°, a small but unambiguously nonzero separation, because the molecule vector of Equation (2) sums substructure embeddings *with multiplicity*. A missing sugar unit removes a definite contribution from the sum and displaces the vector accordingly. That the same mechanism produces the same qualitative failure of the fingerprint in two chemically unrelated settings—a salicylate oligomer series of *M*_W_ < 700 Da and a cardiac‐glycoside homolog of *M*_W_ ≈ 765 Da—suggests it is a general property of binary substructure fingerprints applied to repeat‐unit series, and one of the clearest arguments for a multiplicity‐aware representation.

We note explicitly that this is *not* a stereochemical effect. The pretrained Mol2Vec model employed here derives its identifiers from nonchiral Morgan environments and would assign two genuine stereoisomers identical vectors, hence *d*_M _= 0; resolving stereochemistry is a limitation of the present representation, not a capability of it.The remaining TSI neighbors (**DT7**–**DT10**) settle into the tie‐heavy pattern described above, with **DT9** and **DT10** sharing TSI = 0.9067 along with an additional molecule outside the five shown.

#### 4.4.4 Cross‐Compound Consistency

Table 1 places the four ACI‐library reference molecules side by side, and two patterns hold across all of them. First, the Tanimoto top‐10 neighbor list collapses to between 3 and 7 distinct scores irrespective of the query, which indicates that the ties originate in the binary representation itself rather than in anything peculiar to aspirin. Second, MSI resolves more distinct values than TSI on every query, between 7 and 10 out of 10, with the largest margin for ibuprofen, whose neighborhood is dominated by alkoxy‐chain homologs—exactly the repeat‐unit situation in which a set‐based fingerprint is least informative. Digitoxin marks the boundary of the effect rather than an exception to it: MSI's resolving power falls to 8/10 because of genuine regiochemical near‐duplicates that the embedding does not separate, yet it still exceeds the 7/10 obtained with the fingerprint on the same query.

Together with the aniline/QM9 comparison of Section 4.3, the evaluation now rests on five reference compounds—aspirin, ibuprofen, curcumin, aniline, and digitoxin—spanning a fourfold range in molecular weight and four distinct structural classes.

### 4.5 Robustness to the Choice of Molecular Representation

Because similarity outcomes can depend as strongly on the chosen molecular representation as on the similarity coefficient itself (Section 2), the benchmarking of Sections 4.2–4.4 was repeated with alternative fingerprint parametrizations, and three additional representation/metric baselines were computed to help separate the contribution of the embedding from that of the Mahalanobis covariance correction. All results below were obtained on the exact molecule sets already used for the aspirin (ACI, *N *= 31 393) and aniline (QM9, *N *= 127 537) case studies, so they are directly comparable to the TSI values reported in Sections 4.2.2 and 4.3.

#### 4.5.1 Fingerprint Parametrization

TSI values were recomputed with ECFP6 fingerprints (Morgan radius 3, 2048 bits) and with ECFP4 folded to 1024 instead of 2048 bits, and compared against the original ECFP4/2048 configuration used throughout the rest of the manuscript. Table 2 reports the Spearman rank correlation (*ρ*) between the original TSI ranking and each alternative, computed over the full library. Halving the fingerprint length (bit length 2048 → 1024, radius unchanged) leaves the ranking essentially unaffected (*ρ *= 0.991 for aspirin, *ρ* = 0.943 for aniline), and the top‐10 neighbor sets are identical in composition for both query molecules; only the internal ordering of tied values shifts marginally. Increasing the fingerprint radius (ECFP4 → ECFP6, bit length unchanged) has a larger effect (*ρ* = 0.984 for aspirin, *ρ *= 0.962 for aniline). For aspirin, 8 of the 9 nonreference neighbors from Figure 3 are retained under ECFP6: the five‐way tie at TSI = 0.8571 occupying the first plateau (**BZ1**, **BZ10**–**BZ13**) is only partially broken, collapsing into a unique top match (the acetylsalicyloyl dimer) followed by a four‐way tie among the remaining oligomers, while **BZ15**–**BZ17** persist near the bottom of the top‐10; only **BZ14** drops out, displaced by an aromatic carbonate‐type isomer absent from the original ECFP4 top‐10. For aniline the effect is more pronounced: only three of the nine nonreference neighbors from Figure 8 survive under ECFP6. The phenylenediamine isomers **NH8**, **NH3**, and **NH10** keep their relative order, but the aminotropones **NH11**–**NH13** and the monosubstituted anilines **NH9**, **NH14**, **NH15** are all displaced from the top‐10 by amino‐free monosubstituted benzenes (phenol, fluorobenzene, toluene, cumene), an additional methylaniline isomer, and benzamide. In neither case is the top match affected: the query molecule itself remains the unique neighbor at TSI = 1 under every parametrization tested. Tables 3 and 4 list the corresponding top‐10 molecules and their TSI values under both configurations for direct comparison.

**TABLE 2:** Spearman rank correlation (*ρ*) between the baseline TSI (ECFP4, radius 2, 2048 bits) and each alternative fingerprint parametrization or molecular representation, computed over the full reference library for each query molecule.

Comparison vs. ECFP4/2048 TSI	Aspirin (*N *= 31 393)	Aniline (*N *= 127 537)
ECFP6/2048	0.984	0.962
ECFP4/1024	0.991	0.943
MACCS + Tanimoto	0.630	0.456
Cosine, 300D Mol2Vec	0.616	0.617
Euclidean, 300D Mol2Vec	0.607	0.609

**TABLE 3:** Top‐10 nearest neighbors of aspirin by Tanimoto similarity, ECFP4 (original) versus ECFP6.

Rank ECFP4	Rank ECFP6	Label	TSI (ECFP4)	TSI (ECFP6)
1	1	Aspirin	1.0000	1.0000
2	5	**BZ10**–**BZ13**	0.8571	0.7143
3	2	**BZ1**	0.8571	0.7317
4	4	**BZ10**–**BZ13**	0.8571	0.7143
5	6	**BZ10**–**BZ13**	0.8571	0.7143
6	3	**BZ10**–**BZ13**	0.8571	0.7143
7	—	**BZ14**	0.7407	0.6667
8	8	**BZ15**–**BZ17**	0.7241	0.6842
9	10	**BZ15**–**BZ17**	0.7241	0.6667
10	7	**BZ15**–**BZ17**	0.7241	0.6842
—	9	—	0.7037	0.6667

*Note:* Rank columns are blank where a molecule falls outside the top‐10 for that configuration, but its TSI value is reported regardless. Labels were resolved by matching canonical SMILES against the reference **BZ1**–**BZ9** set (Mahalanobis top‐9) and the TSI values quoted in Section 4.2.2: **BZ1** (the acetylsalicyloyl dimer) and **BZ14** are each pinned down uniquely this way. The remaining four molecules tied at TSI = 0.8571 are jointly labeled **BZ10**–**BZ13**, and the three tied at TSI = 0.7241 are jointly labeled **BZ15**–**BZ17** because their individual correspondence could not be established: these four‐/three‐way ties are internal to the TSI ranking itself and are not resolved by the **BZ1**–**BZ9** Mahalanobis‐ranking reference set.

**TABLE 4:** Top‐10 nearest neighbors of aniline by Tanimoto similarity, ECFP4 (original) versus ECFP6.

Rank ECFP4	Rank ECFP6	Label	TSI (ECFP4)	TSI (ECFP6)
1	1	Aniline	1.0000	1.0000
2	2	**NH8**	0.5833	0.4375
3	3	**NH3**	0.5333	0.4211
4	4	**NH10**	0.5000	0.3889
5	—	**NH11**/**NH12**	0.4737	0.3214
6	—	**NH11**/**NH12**	0.4737	0.3214
7	—	**NH13**	0.4211	0.3333
8	—	**NH9**	0.4118	0.3182
9	—	**NH15**	0.4118	0.3182
10	—	**NH14**	0.4118	0.3182
—	5	**NH4**	0.3750	0.3684
—	6	—	0.3750	0.3684
—	7	**NH1**	0.3750	0.3684
—	8	—	0.3889	0.3636
—	9	**NH2**	0.4000	0.3333
—	10	—	0.3500	0.3333

*Note:* Rank columns are blank where a molecule falls outside the top‐10 for that configuration, but its TSI value is reported regardless. Labels were resolved by matching canonical SMILES against the reference **NH1**–**NH9** set (Mahalanobis top‐9) and the substituent classes quoted in Section 4.3 (phenylenediamines **NH3**/**NH8**/**NH10**; aminophenols **NH6**/**NH14**; aminotropones **NH11**–**NH13**; halogenated aniline **NH15**). **NH11** and **NH12** remain jointly labeled because the tie between the two aminotropone isomers at TSI = 0.4737 is internal to the TSI ranking and not resolved by the **NH1**–**NH9** reference set.

#### 4.5.2 Ablations

Three further baselines were computed to test whether the Mahalanobis correction, and not merely the switch from a binary circular fingerprint to a continuous embedding, is responsible for the ranking behavior documented throughout this work: (i) MACCS structural keys (166 bits, nonhashed, noncircular) with the Tanimoto coefficient; (ii) cosine similarity computed directly on the Mol2Vec embeddings used by MSI (Σ = *I*, i.e., no covariance weighting); and (iii) Euclidean distance on the same embeddings, the direct Σ = *I* contrast to Equation (3) . All three correlate only moderately with the ECFP4/2048 TSI ranking (Table 2; *ρ* between 0.46 and 0.63 across both reference molecules), and their top‐10 neighbor lists overlap with the TSI top‐10 by just 1–6 out of 10 molecules, confirming that TSI and these alternative representations/metrics are not interchangeable rankings of the same chemical neighborhood. MACCS keys are notably coarser than the Morgan fingerprints for aspirin: eight molecules share the maximum MACCS Tanimoto value of 1.0 with the aspirin query—versus a unique top match under every ECFP configuration tested—reflecting the lower resolution of a 166‐bit, noncircular key set. Restricting the comparison to *d*_M_ itself (rather than TSI) gives a similar picture for aspirin, where cosine similarity on the raw embedding correlates with *d*_M_ at *ρ* = − 0.423; for aniline this correlation is weak and of the opposite sign (*ρ *= +0.327), consistent with the two‐regime, largely flat relationship between *d*_M_ and similarity already noted for the QM9 library in Section 4.3, where most of the 127 537 molecules occupy the low‐similarity basin and only the immediate neighborhood of aniline carries an appreciable signal. Together, these ablations indicate that neither a coarser binary key set nor an uncorrected continuous embedding reproduces MSI's ranking behavior on its own, though disentangling the specific contribution of the covariance correction from that of the embedding more rigorously would require a dedicated study beyond the scope of this robustness check.

We note, finally, that the Mol2Vec embeddings themselves (and therefore *d*_M_ and *θ*_M_) are not recomputed here with an alternative substructure‐extraction radius: mol2alt_sentence radius is tied to the vocabulary of the pretrained Word2Vec model (model_300dim.pkl), and varying it would require retraining that model, which lies outside the scope of the present check. The parametrizations varied above instead target the external TSI benchmark against which MSI is compared throughout the manuscript.

### 4.6 Scope and Limitations

Five limitations bound the conclusions that can be drawn from this work, and we state them together rather than distributing them through the discussion.

*The angular coordinate is largely redundant with the radial one*. As shown in Equation (6), *d*_M_ and *θ*_M_ are functionally related whenever the whitened norms of the library are approximately constant, and the observed Spearman correlation of *ρ *= 1.0000 (Table 1) indicates that this is the case here. The “dual radial–angular” description should therefore be read as a convenience—*θ*_M_ supplies a bounded, dimension‐insensitive scale on which to set thresholds—and not as a claim that the angle resolves molecules the distance cannot.

*The evaluation is a retrieval and ranking comparison*, *not a screening benchmark*. No enrichment factors, ROC curves, or active/decoy analyses are reported because the libraries used are analog collections assembled by substructure search rather than annotated bioactivity sets. The advantage of MSI documented here is a reduction in ranking ties and a more chemically interpretable neighborhood; it is not a demonstration of improved hit rates in virtual screening. Establishing the latter requires a prospective evaluation on a standard benchmark such as DUD‐E or LIT‐PCBA and is the natural next step.

*The representation is blind to stereochemistry and weakly sensitive to positional isomerism*. The pretrained Mol2Vec model derives nonchiral Morgan identifiers, so stereoisomers are assigned identical vectors. Positional isomers of small substituents on a conserved scaffold are separated only weakly, as the curcumin near‐duplicates (Section 4.4) and the digitoxin acetoxy variants (Section 4.4.3) both show. Where these distinctions matter, a fingerprint or a 3D descriptor is the better representation, and the two are complementary rather than competing.

*Results are conditional on the library used to estimate* Σ. The covariance matrix is estimated separately for each library, so *d*_M_ values are comparable within a library but not between libraries, and the applicability domains quoted in the text should be rederived whenever the reference collection changes. The dependence of MSI rankings on library composition has not been characterized systematically.

*Activity cliffs remain outside the scope of any similarity index*. As discussed in Section 4.3, the finer discrimination that MSI provides does not by itself predict whether a given structural perturbation will produce a discontinuity in activity.

## 5 Conclusions

We introduce the Mol2Vec–MSI, a computational framework that couples continuous vector embeddings with Mahalanobis distance and angular metrics to overcome systematic biases in traditional fingerprint‐based (binary feature‐based) molecular similarity assessment.

Evaluation on five reference compounds—aspirin, aniline, curcumin, ibuprofen, and digitoxin—points to three advantages of MSI over the ECFP4 + Tanimoto baseline. First, MSI promotes candidates that the fingerprint systematically deprioritises: toluene ranks first by Mahalanobis distance but only ninth by TSI for aniline because a binary fingerprint penalizes molecules lacking a specific functional group even when the underlying electronic character is preserved—a distinction that matters for scaffold hopping. Second, MSI resolves Tanimoto ties. Structurally distinct molecules such as methylated, hydroxylated, and halogenated anilines frequently receive identical Tanimoto scores, and the effect is at its most extreme for repeat‐unit series: salicylate oligomers and the *bis*‐digitoxoside homolog of digitoxin are scored 0.8571 and 1.0000 respectively, indistinguishably from far closer analogs because a set‐based representation records the presence of a substructure but not its multiplicity. The multiplicity‐weighted Mol2Vec sum does not share this blind spot. Third, MSI geometry varies systematically with the HOMO–LUMO gap: within *d*_M _< 25 and *θ*_M _< 10° the QM9 candidates show comparable frontier‐orbital characteristics, and the gap broadens outside that envelope. Since the embedding was trained on structural context alone, this correspondence is emergent rather than fitted, which makes it a meaningful indication that the geometry captures chemistry and not an artifact of the representation.

Polar plots and 3D similarity maps show that dissimilarity mainly increases in certain descriptor directions, forming “chemical corridors” for analog discovery. In the QM9 set near aniline, 40% of molecules cluster in regions with Mahalanobis distance *d*_M _< 25 and direction angle *θ*_M _< 10°, while sparse outer areas (*d*_M _> 100, *θ*_M _> 45°) offer opportunities for scaffold hopping. These maps define natural applicability domains: strict thresholds (*d*_M _≤ 20, *θ*_M _≤ 5°) select very similar analogs with similar structures and functions, while moderate thresholds (*d*_M _≤ 50, *θ*_M _≤ 15°) blend similarity with diversity for lead optimization. Mahalanobis metric remains useful even when the TSI becomes uninformative near zero.

The aspirin study validated MSI on pharmaceutically relevant molecules. Top analogs preserved the salicylate pharmacophore with incremental modifications reflected as modest *d*_M_ increases and consistent angular alignment (*θ*_M _≈ 0.8°–1.1°). In contrast, TSI produced multiple five‐way ties at identical scores despite meaningful structural differences. Building on these findings, the aniline study extended validation to optoelectronically significant aromatics. Here, MSI rankings correlated strongly with substituent electronic effects (electron‐donating groups: negative Δ*E*_gap_ shifts; electron‐withdrawing: positive shifts), while TSI rankings showed poor correspondence. The aminotropone isomers, for example, showed the largest reductions of the HOMO–LUMO gap yet received only moderate Tanimoto scores, whereas phenylenediamines achieved top Tanimoto ranks despite substantial electronic deviation from aniline.

LOWESS analysis revealed universal two‐regime behavior: steep similarity decay within the inner Mahalanobis envelope and then plateau formation. The high‐gradient region efficiently identifies close analogs; the plateau region contains molecules with residual correlations that Mahalanobis geometry detects but fingerprints miss—valuable leads for diversification.

Similarity metric choice should align with screening objectives. TSI is suitable for broad chemotype retrieval in fragment identification. MSI excels when objectives emphasize property matching (biological activity, ADMET, reactivity) through continuous, property‐aware representations. The systematic rank disagreements reflect fundamental differences: TSI measures substructural inventory; MSI assesses position in a learned covariance‐aware physicochemical space.

Future extensions include integration with quantum‐chemical descriptors, multiobjective formulations combining Mahalanobis geometry with synthetic‐accessibility or toxicity filters, and prospective virtual‐screening validation on annotated benchmarks. Section 4.4 adds curcumin, ibuprofen, and digitoxin as reference molecules, and the same pattern recurs for all three: the Tanimoto top‐ranked hits collapse into a handful of tied scores, while MSI separates most or all of them. Digitoxin also delimits the effect since it shows that MSI's tie‐breaking is bounded by what the Mol2Vec embedding resolves, and for near‐identical regiochemical variants it does not fully succeed—a limitation we prefer to state explicitly rather than report only the favorable cases. The descriptor ablations reported in Section 4.5 show that MACCS keys, and cosine or Euclidean similarity computed on the same Mol2Vec embedding without the covariance correction, all correlate only moderately with TSI (*ρ *= 0.46–0.63 across the two reference libraries) and recover just 1–6 of the original 10 TSI‐ranked neighbors, indicating that neither a coarser binary key set nor an uncorrected continuous embedding reproduces MSI's ranking behavior on its own—though a full decomposition of the covariance correction's specific contribution is left to future work. Taken together, the five reference compounds studied here—two NSAIDs, a polyphenolic natural product, an industrial aromatic amine, and a cardiac glycoside, spanning 93–765 Da—support the same conclusion across a considerably wider structural range than the original aspirin/aniline pair.

The Mol2Vec–MSI provides a statistically grounded, geometrically interpretable way to navigate high‐dimensional chemical space. By removing the dependence on fingerprint bit density, retaining information about fragment multiplicity, resolving ranking ties, and tracking physicochemical relationships that the binary representation does not encode, MSI offers a complementary view to fingerprint‐based searching rather than a replacement for it. Methods that pair learned representations with chemically interpretable metrics, of which MSI is one example, seem well positioned to help turn large chemical databases into concrete therapeutic and technological leads.

## Author Contributions

This work represents a collaborative effort among all authors, described below following the CRediT taxonomy. **Roberto Bernal‐Jaquez:** conceptualization, methodology, formal analysis, supervision, writing – review and editing. **Elliot Ridout‐Buhl:** software, visualization, data curation, investigation. **Emiliano Montoya:** software, data curation, validation. **León Alday‐Toledo:** data curation, visualization, investigation, validation, writing – review and editing. **Felipe Aparicio:** investigation, resources, writing – original draft, methodology, writing – review and editing. All authors participated in the interpretation of the results, provided critical feedback, and approved the final manuscript.

## Funding

This work was supported by the Universidad Autónoma Metropolitana (127 S289‐25).

## Conflicts of Interest

The authors declare no conflicts of interest.

## Supporting information

Supplementary Material

Supplementary Material

Supplementary Material

## Data Availability

All source code, input files, and Jupyter notebooks required to reproduce the analyses reported here are available under the MIT license at https://github.com/JACKNINES/MSI. The curated SMILES lists defining the ACI and digitoxin‐augmented libraries are included in the same repository. QM9 [30] and PubChem [29] are publicly available from their respective providers.
