# Supplementary material for: MSI: A Mahalanobis‐Based Molecular Similarity Index for High‐Dimensional Embeddings
Source: Mol Inform. 2026 Aug 27;45(9):e70051. doi: 10.1002/minf.70051 (PMC13522730; doi:10.1002/minf.70051)
Supplement: Supplementary file 1 [file MINF-45-e70051-s001.pdf]

Supporting Information Part II
MSI: A Mahalanobis-Based Molecular Similarity Index for High-Dimensional Embeddings

Roberto Bernal-Jaquez,[*,†] Elliot Ridout-Buhl,[‡] Emiliano Montoya,[¶] León Alday-Toledo,[¶] and Felipe Aparicio[*,‡]

†*Departamento de Matemáticas Aplicadas y Sistemas, División de Ciencias Naturales e Ingeniería, Universidad Autónoma Metropolitana, Unidad Cuajimalpa, Mexico City, Mexico.*

‡*Departamento de Ciencias Naturales, División de Ciencias Naturales e Ingeniería, Universidad Autónoma Metropolitana, Unidad Cuajimalpa, Mexico City, Mexico.*

¶*Posgrado en Ciencias Naturales e Ingeniería, División de Ciencias Naturales e Ingeniería, Universidad Autónoma Metropolitana, Unidad Cuajimalpa, Mexico City, Mexico.*

E-mail: rbernal@cua.uam.mx; fplatas@cua.uam.mx

Technical explanation and improvements

This work presents an improved K-means based clustering algorithm, specifically designed for molecular data sets. This process implies a high computational cost, articularly for datasets where variable relationships are pivotal, however, we sought to optimize complex calculations using parallelization. The tools and improvements made to the code are explained below.

Before clustering, the data is normalized using the *StandardScaler* method, ensuring a mean of zero and a unit standard deviation. This preprocessing step is crucial to mitigate scale biases that might arise from variables with disparate ranges. Furthermore, normalization facilitates faster convergence since Euclidean distances, which K-means relies on, are highly sensitive to variable units. This ensures a robust and efficient clustering process, particularly for datasets with heterogeneous scales.

The initialization of centroids employs the K-means++ strategy, which significantly improves both convergence speed and clustering quality compared to purely random initialization. By selecting initial centroids probabilistically, with a preference for dispersed points, this method balances exploration and exploitation. It reduces the number of iterations required for the algorithm to converge, providing a more stable starting point for the optimization of cluster centroids.

The inclusion of Mahalanobis distance instead of the traditional Euclidean metric introduces a significant enhancement, particularly for datasets where variable correlations are critical. Mahalanobis distance accounts for these correlations, enabling more accurate clustering in non-isotropic data spaces. Although computationally more intensive, this choice is well-suited for molecular data analysis, where inter-variable relationships are essential. Parallelization using `joblib.Parallel` is implemented to enhance computational efficiency. By parallelizing the computation of Mahalanobis distances for each data point, the workload is effectively distributed across multiple cores. This approach drastically reduces processing times for medium to large datasets, particularly in systems equipped with multi-core processors, without compromising the accuracy of the results.

The MK2 method introduces a dynamic centroid selection process, guided by a weighting parameter w. This approach prioritizes points with lower cumulative distances, ensuring that the initial centroids are well-distributed. While not as sophisticated as hierarchical clustering preselection, this practical solution effectively mitigates poor initializations, enhancing clustering outcomes.

Angles

The angles module integrates mathematical and cheminformatics techniques to analyze molecular datasets. The implementation is based on Python, leveraging robust libraries such as `RDKit` for molecular representations and `SciPy` for computational distance metrics. This module is focused on the followinf aspects:

Matrix Operations for Mahalanobis Calculations

The calculation of the Mahalanobis distance and angle relies on linear algebra operations:

- **Covariance Matrix Computation:** The covariance matrix Σ of molecular embeddings is estimated using `NumPy's cov` function. To ensure numerical stability, the matrix is regularized by adding a small value ϵ to its diagonal:

$$\Sigma \longleftarrow \Sigma + \epsilon \cdot I \tag{0.1}$$

  This step avoids issues with singular matrices during inversion.

- **Matrix Inversion:** The inverse covariance matrix Σ^{-1} is computed using `NumPy's linalg.inv`, a computationally intensive operation with a time complexity of $O(n^3)$ for $n \times n$ matrices. To mitigate this cost, the module processes datasets in chunks for large-scale data.

- **Dot Products:** Key calculations, such as inner products and norms, are performed

using NumPy's highly optimized dot function, which leverages `SIMD` (Single Instruction, Multiple Data) and `BLAS` (Basic Linear Algebra Subprograms) for performance.

Vectorized Operations for DataFrame Processing

The module makes extensive use of pandas and NumPy to vectorize operations:

- **Row-wise Mahalanobis Distance:** Distances are calculated for each row in a molecular embedding matrix by applying vectorized functions. This avoids Python loops and takes advantage of low-level optimizations in `NumPy`.

Fingerprinting and Similarity Computation

The Tanimoto similarity relies on molecular fingerprints generated via RDKit's ECFP algorithm:

- **ECFP Generation:** Morgan fingerprints are created with a radius of 2 and a fixed size of 2048 bits. This process involves hashing molecular substructures and is efficient even for large datasets due to RDKit's optimized C++ backend.

- **Bitwise Operations:** The Tanimoto coefficient is computed using efficient bitwise operations over the binary vectors, reducing computational overhead.

Against-One Analysis: Computational Workflow

The against one function aligns two datasets: one containing molecular descriptors and another containing molecular embeddings. Key computational steps include:

1. **Covariance Matrix Regularization:** Ensures invertibility and stability during distance calculations.

2. **Reference Vector Selection:** The first row in the embedding dataset is treated as the reference vector for all similarity and distance computations.

3. **SMILES Conversion:** Molecular SMILES strings are converted to RDKit Mol objects for fingerprint generation and similarity calculations.

Future Improvements

Despite its current efficiency and accuracy, the method presents several avenues for future optimization and development. First, the computational cost of Mahalanobis distance could be further reduced by integrating distributed computing frameworks, allowing the inversion of the covariance matrix and distance calculations to scale efficiently with dataset size. Additionally, adaptive dimensionality reduction techniques could be explored to dynamically balance computational complexity and accuracy, particularly in datasets with highly redundant or irrelevant features.

For the MK2 method, a promising direction would involve incorporating data-driven approaches to determine the weighting parameter w. Machine learning models could be employed to predict optimal weights based on dataset characteristics, enhancing the robustness of centroid selection. Moreover, hybrid approaches combining MK2 with other initialization strategies, such as hierarchical clustering or density-based methods, could provide more consistent results across diverse datasets.
