# Supplementary material for: MSI: A Mahalanobis‐Based Molecular Similarity Index for High‐Dimensional Embeddings
Source: Mol Inform. 2026 Aug 27;45(9):e70051. doi: 10.1002/minf.70051 (PMC13522730; doi:10.1002/minf.70051)
Supplement: Supplementary file 2 [file MINF-45-e70051-s002.pdf]

Supporting Information: Full Names of Analog Molecules

This document lists the full chemical names corresponding to the short labels (**BZ**n, **NH**n, **CU**n, **IB**n, **DT**n) used throughout the main text and figures to identify analog molecules retrieved for each of the five reference compounds studied (aspirin, aniline, curcumin, ibuprofen, and digitoxin).

Aspirin analogs (BZ series)

**Label**	**Compound name**
BZ1	2-((2-acetoxybenzoyl)oxy)benzoic acid
BZ2	2-((2,5-diacetoxybenzoyl)oxy)benzoic acid
BZ3	2-acetoxy-5-(benzoyloxy)benzoic acid
BZ4	2,5-diacetoxybenzoic acid
BZ5	2,4-diacetoxybenzoic acid
BZ6	2-acetoxy-5-methylbenzoic acid
BZ7	2-acetoxy-4-methylbenzoic acid
BZ8	2,5-diacetoxy-terephthalic acid
BZ9	4,6-diacetoxyisophthalic acid
BZ10	2-((2-((2-((2-((2-acetoxybenzoyl)oxy)benzoyl)oxy)benzoyl)oxy) benzoyl)oxy)benzoic acid
BZ11	2-((2-((2-((2-acetoxybenzoyl)oxy)benzoyl)oxy)benzoyl)oxy)ben zoic acid
BZ12	2-((2-((2-((2-((2-((2-acetoxybenzoyl)oxy)benzoyl)oxy)benzoyl) oxy)benzoyl)oxy)benzoyl)oxy)benzoic acid
BZ13	2-((2-((2-acetoxybenzoyl)oxy)benzoyl)oxy)benzoic acid
BZ14	2-acetylphenyl acetate
BZ15	2-(prop-1-en-2-yloxy)benzoic acid
BZ16	2-(ethanethioyloxy)benzoic acid
BZ17	2-((2-oxopropanoyl)oxy)benzoic acid

Aniline analogs (NH series)

**Label**	**Compound name**
NH1	Toluene
NH2	*m*-Toluidine (3-methylaniline)
NH3	Benzene-1,3-diamine (*m*-phenylenediamine)
NH4	Phenol
NH5	Ethylbenzene
NH6	3-Aminophenol
NH7	Anisole (methoxybenzene)
NH8	Benzene-1,4-diamine (*p*-phenylenediamine)
NH9	*p*-Toluidine (4-methylaniline)
NH10	Benzene-1,2-diamine (*o*-phenylenediamine)
NH11	Aminocycloheptatrienone isomer (regiochemistry not specified in the manuscript text — please confirm)

Label	Compound name
NH12	Aminocycloheptatrienone isomer (regiochemistry not specified in the manuscript text — please confirm)
NH13	Aminocycloheptatrienone isomer (regiochemistry not specified in the manuscript text — please confirm)
NH14	4-Aminophenol
NH15	4-Fluoroaniline

Curcumin analogs (CU series)

Label	Compound name
CU1	(1*E*,6*E*)-1-(4-hydroxy-3-methoxyphenyl)-7-(3-hydroxy-4-methoxyphenyl)hepta-1,6-diene-3,5-dione
CU2	(1*E*,6*E*)-1,7-bis(3-hydroxy-4-methoxyphenyl)hepta-1,6-diene-3,5-dione
CU3	4-((1*E*,6*E*)-7-(4-hydroxy-3-methoxyphenyl)-3,5-dioxohepta-1,6-dien-1-yl)-2-methoxyphenyl 2-hydroxybenzoate
CU4	(1*E*,1'*E*,6*E*,6'*E*)-7,7'-((ethane-1,2-diylbis(oxy))bis(4-hydroxy-3,1-phenylene))bis(1-(4-hydroxy-3-methoxyphenyl)hepta-1,6-diene-3,5-dione)
CU5	(1*E*,6*E*)-1-(3-(hexyloxy)-4-hydroxyphenyl)-7-(4-hydroxy-3-methoxyphenyl)hepta-1,6-diene-3,5-dione
CU6	(1*E*,6*E*)-1-(3,4-dihydroxyphenyl)-7-(4-hydroxy-3-methoxyphenyl)hepta-1,6-diene-3,5-dione
CU7	(1*E*,6*E*)-1-(3,4-dimethoxyphenyl)-7-(4-hydroxy-3-methoxyphenyl)hepta-1,6-diene-3,5-dione
CU8	(*E*)-6-(4-hydroxy-3-methoxyphenyl)hex-5-ene-2,4-dione
CU9	(*E*)-6-(4-hydroxy-3-methoxyphenyl)-2,4-dioxohex-5-enal
CU10	(1*E*,6*E*)-1-(4-hydroxy-3,5-dimethoxyphenyl)-7-(4-hydroxy-3-methoxyphenyl)hepta-1,6-diene-3,5-dione

Ibuprofen analogs (IB series)

Label	Compound name
IB1	2-(3-isobutylphenyl)propanoic acid
IB2	2-(4-isobutyl-3-methylphenyl)propanoic acid
IB3	2-((2-(4-isobutylphenyl)propanoyl)oxy)benzoic acid
IB4	2-(3-fluoro-4-isobutylphenyl)propanoic acid
IB5	2-(4-isobutyl-2-methylphenyl)propanoic acid
IB6	2-(4'-isobutyl-[1,1'-biphenyl]-4-yl)propanoic acid
IB7	3-(4-(1-carboxyethyl)phenyl)-2-methylpropanoic acid
IB8	2-(4-(2-hydroxypropyl)phenyl)propanoic acid
IB9	2-(4-(2,4-dimethylpentyl)phenyl)propanoic acid
IB10	2-(4-(2,2-difluoroethyl)phenyl)propanoic acid

Digitoxin analogs (DT series)

Label	Compound name
DT1	4-((3*S*,5*R*,10*S*,13*R*,14*S*,17*R*)-14-hydroxy-3-(((2*R*,4*S*,5*S*,6*R*)-4-hydroxy-5-(((2*S*,4*S*,5*S*,6*R*)-4-hydroxy-5-(((2*R*,5*R*,6*S*)-5-hydroxy-6-methyltetrahydro-2*H*-pyran-2-yl)oxy)-6-methyltetrahydro-2*H*-pyran-2-yl)oxy)-6-methyltetrahydro-2*H*-pyran-2-yl)oxy)-10,13-dimethylhexadecahydro-1*H*-cyclopenta[*a*]phenanthren-17-yl)furan-2(5*H*)-one
DT2	4-hydroxy-6-((4-hydroxy-6-((4-hydroxy-6-((14-hydroxy-10,13-dimethyl-17-(5-oxo-2,5-dihydrofuran-3-yl)hexadecahydro-1*H*-cyclopenta[*a*]phenanthren-3-yl)oxy)-2-methyltetrahydro-2*H*-pyran-3-yl)oxy)-2-methyltetrahydro-2*H*-pyran-3-yl)oxy)-2-methyltetrahydro-2*H*-pyran-3-yl acetate
DT3	4-(14-hydroxy-3-((4-hydroxy-5-((4-hydroxy-5-((4-hydroxy-5-methoxy-6-methyltetrahydro-2*H*-pyran-2-yl)oxy)-6-methyltetrahydro-2*H*-pyran-2-yl)oxy)-6-methyltetrahydro-2*H*-pyran-2-yl)oxy)-10,13-dimethylhexadecahydro-1*H*-cyclopenta[*a*]phenanthren-17-yl)furan-2(5*H*)-one
DT4	(2*R*,3*R*,4*S*,6*S*)-3-hydroxy-6-(((2*R*,3*S*,4*S*,6*S*)-4-hydroxy-6-(((2*R*,3*S*,4*S*,6*R*)-4-hydroxy-6-(((3*S*,5*R*,8*R*,9*S*,10*S*,13*R*,14*S*,17*R*)-14-hydroxy-10,13-dimethyl-17-(5-oxo-2,5-dihydrofuran-3-yl)hexadecahydro-1*H*-cyclopenta[*a*]phenanthren-3-yl)oxy)-2-methyltetrahydro-2*H*-pyran-3-yl)oxy)-2-methyltetrahydro-2*H*-pyran-3-yl)oxy)-2-methyltetrahydro-2*H*-pyran-4-yl acetate
DT5	(2*R*,3*S*,4*S*,6*R*)-6-(((2*R*,3*R*,4*S*,6*S*)-4-(((2*R*,4*S*,5*S*,6*R*)-4,5-dihydroxy-6-methyltetrahydro-2*H*-pyran-2-yl)oxy)-6-(((3*S*,5*R*,8*R*,9*S*,10*S*,13*R*,17*R*)-14-hydroxy-10,13-dimethyl-17-(5-oxo-2,5-dihydrofuran-3-yl)hexadecahydro-1*H*-cyclopenta[*a*]phenanthren-3-yl)oxy)-2-methyltetrahydro-2*H*-pyran-3-yl)oxy)-4-hydroxy-2-methyltetrahydro-2*H*-pyran-3-yl acetate
DT6	4-((3*S*,5*R*,8*R*,9*S*,10*S*,13*R*,14*S*,17*R*)-3-(((2*R*,4*S*,5*S*,6*R*)-5-(((2*S*,4*S*,5*S*,6*R*)-4,5-dihydroxy-6-methyltetrahydro-2*H*-pyran-2-yl)oxy)-4-hydroxy-6-methyltetrahydro-2*H*-pyran-2-yl)oxy)-14-hydroxy-10,13-dimethylhexadecahydro-1*H*-cyclopenta[*a*]phenanthren-17-yl)furan-2(5*H*)-one
DT7	4-((3*S*,5*R*,8*R*,9*S*,10*S*,13*R*,14*S*,17*R*)-3-(((2*S*,4*S*,5*R*,6*S*)-4,5-dihydroxy-6-methyltetrahydro-2*H*-pyran-2-yl)oxy)-14-hydroxy-10,13-dimethylhexadecahydro-1*H*-cyclopenta[*a*]phenanthren-17-yl)furan-2(5*H*)-one

Label	Compound name
DT8	4-((3*S*,5*S*,9*S*,10*S*,13*R*,14*S*,17*R*)-3-(((2*R*,4*S*,5*S*,6*S*)-5-(((2*S*,4*S*,5*S*,6*R*)-5-(((2*S*,4*S*,5*S*,6*R*)-4,5-dihydroxy-6-methyltetrahydro-2*H*-pyran-2-yl)oxy)-4-hydroxy-6-methyltetrahydro-2*H*-pyran-2-yl)oxy)-4,6-dihydroxytetrahydro-2*H*-pyran-2-yl)oxy)-14-hydroxy-10,13-dimethylhexadecahydro-1*H*-cyclopenta[*a*]phenanthren-17-yl)furan-2(5*H*)-one
DT9	4-((3*S*,5*R*,9*S*,10*S*,13*R*,14*S*,17*R*)-3-(((2*R*,4*S*,5*S*,6*R*)-5-(((2*S*,4*S*,5*R*,6*R*)-5-(((2*S*,4*S*,5*S*,6*R*)-4,5-dihydroxy-6-methyltetrahydro-2*H*-pyran-2-yl)oxy)-4-hydroxy-6-methyl-1,3-dioxan-2-yl)oxy)-4-hydroxy-6-methyltetrahydro-2*H*-pyran-2-yl)oxy)-14-hydroxy-10,13-dimethylhexadecahydro-1*H*-cyclopenta[*a*]phenanthren-17-yl)furan-2(5*H*)-one
DT10	4-(3-((5-((5-((4,5-dihydroxy-6-methyltetrahydro-2*H*-pyran-2-yl)oxy)-4-methoxy-6-methyltetrahydro-2*H*-pyran-2-yl)oxy)-4-hydroxy-6-methyltetrahydro-2*H*-pyran-2-yl)oxy)-14-hydroxy-10,13-dimethylhexadecahydro-1*H*-cyclopenta[*a*]phenanthren-17-yl)furan-2(5*H*)-one

Note: Names for NH11–NH13 (aminocycloheptatrienone isomers) could not be individually resolved from the manuscript text, which describes them only collectively; the corresponding author should supply the specific substitution pattern for each isomer before this document is finalized.
