# Supplementary material for: MSI: A Mahalanobis‐Based Molecular Similarity Index for High‐Dimensional Embeddings
Source: Mol Inform. 2026 Aug 27;45(9):e70051. doi: 10.1002/minf.70051 (PMC13522730; doi:10.1002/minf.70051)
Supplement: Supplementary file 3 [file MINF-45-e70051-s003.pdf]

Supporting Information Part I MSI: A Mahalanobis-Based Molecular Similarity Index for High-Dimensional Embeddings

Roberto Bernal-Jaquez,[*,†] Elliot Ridout-Buhl,[‡] Emiliano Montoya,[¶] León Alday-Toledo,[¶] and Felipe Aparicio[*,‡]

†*Departamento de Matemáticas Aplicadas y Sistemas, División de Ciencias Naturales e Ingeniería, Universidad Autónoma Metropolitana, Unidad Cuajimalpa, Mexico City, Mexico.*

‡*Departamento de Ciencias Naturales, División de Ciencias Naturales e Ingeniería, Universidad Autónoma Metropolitana, Unidad Cuajimalpa, Mexico City, Mexico.*

¶*Posgrado en Ciencias Naturales e Ingeniería, División de Ciencias Naturales e Ingeniería, Universidad Autónoma Metropolitana, Unidad Cuajimalpa, Mexico City, Mexico.*

E-mail: rbernal@cua.uam.mx; fplatas@cua.uam.mx

Additional Figures

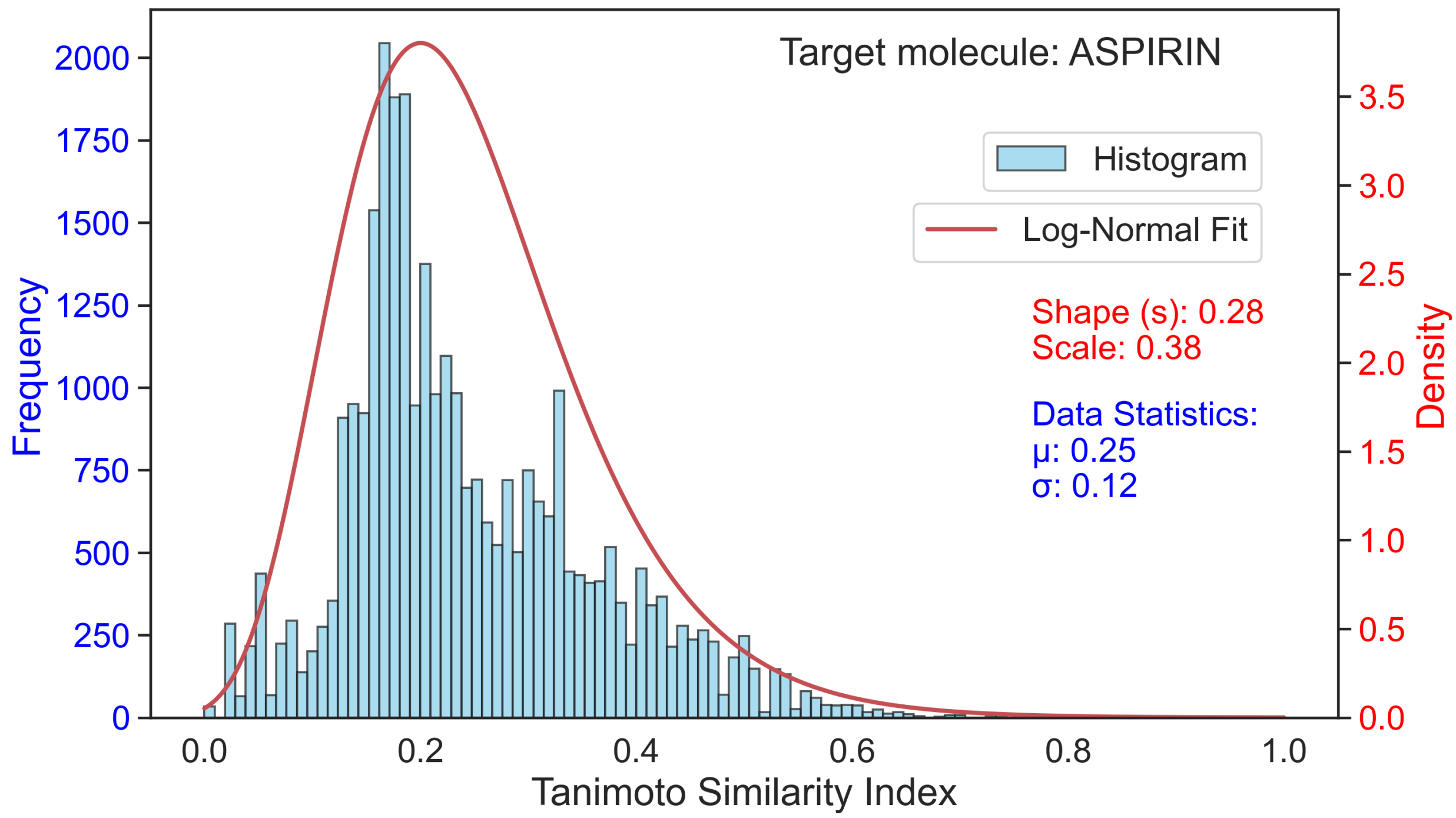

Figure S1. **Distribution of Tanimoto Similarity Index values for the ACI dataset referenced to aspirin.** Blue bars represent the frequency histogram of TSI values across all molecules in the ACI library. The red curve shows the fitted log-normal distribution with shape parameter $s = 0.28$ and scale parameter $= 0.38$. Data statistics indicate mean $\mu = 0.25$ and standard deviation $\sigma = 0.12$. The distribution exhibits positive skewness with maximum frequency near TSI ≈ 0.2, indicating that most molecules in the ACI dataset share low-to-moderate structural similarity with aspirin. The long tail extending toward higher TSI values represents increasingly similar analogs, with few molecules achieving TSI > 0.6.

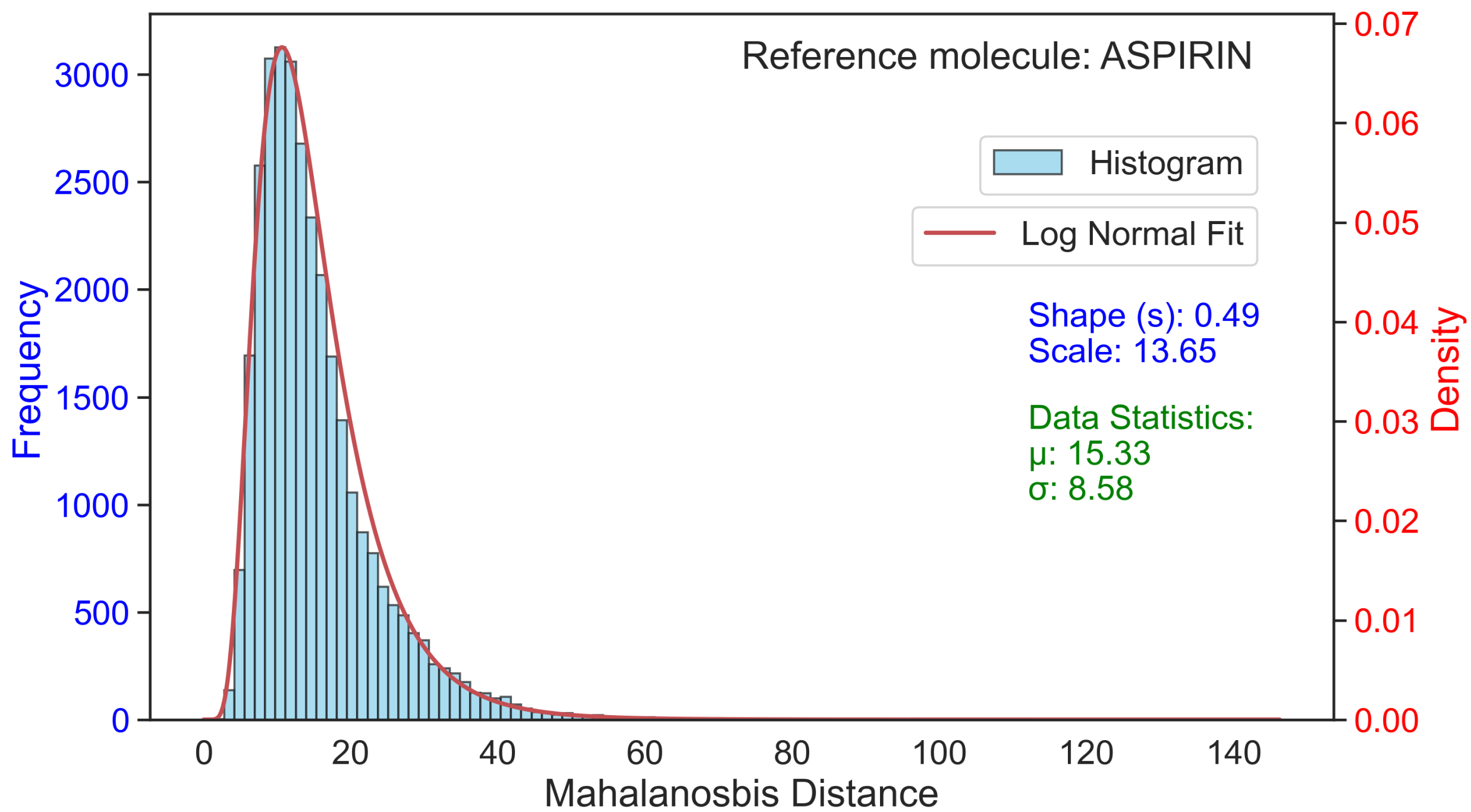

Figure S2. **Distribution of Mahalanobis distance values for the ACI dataset referenced to aspirin.** Blue bars represent the frequency histogram of Mahalanobis distance (d_M) values across all molecules in the ACI library. The red curve shows the fitted log-normal distribution with shape parameter $s = 0.49$ and scale parameter $= 13.65$. Data statistics indicate mean $\mu = 15.33$ and standard deviation $\sigma = 8.58$. The distribution exhibits strong positive skewness with maximum frequency at $d_M \approx 10$–15, indicating that most molecules in the ACI dataset occupy proximate regions of Mahalanobis space relative to aspirin. The extended tail toward larger distances represents increasingly dissimilar molecules in the learned embedding space, with few molecules exceeding $d_M > 60$.

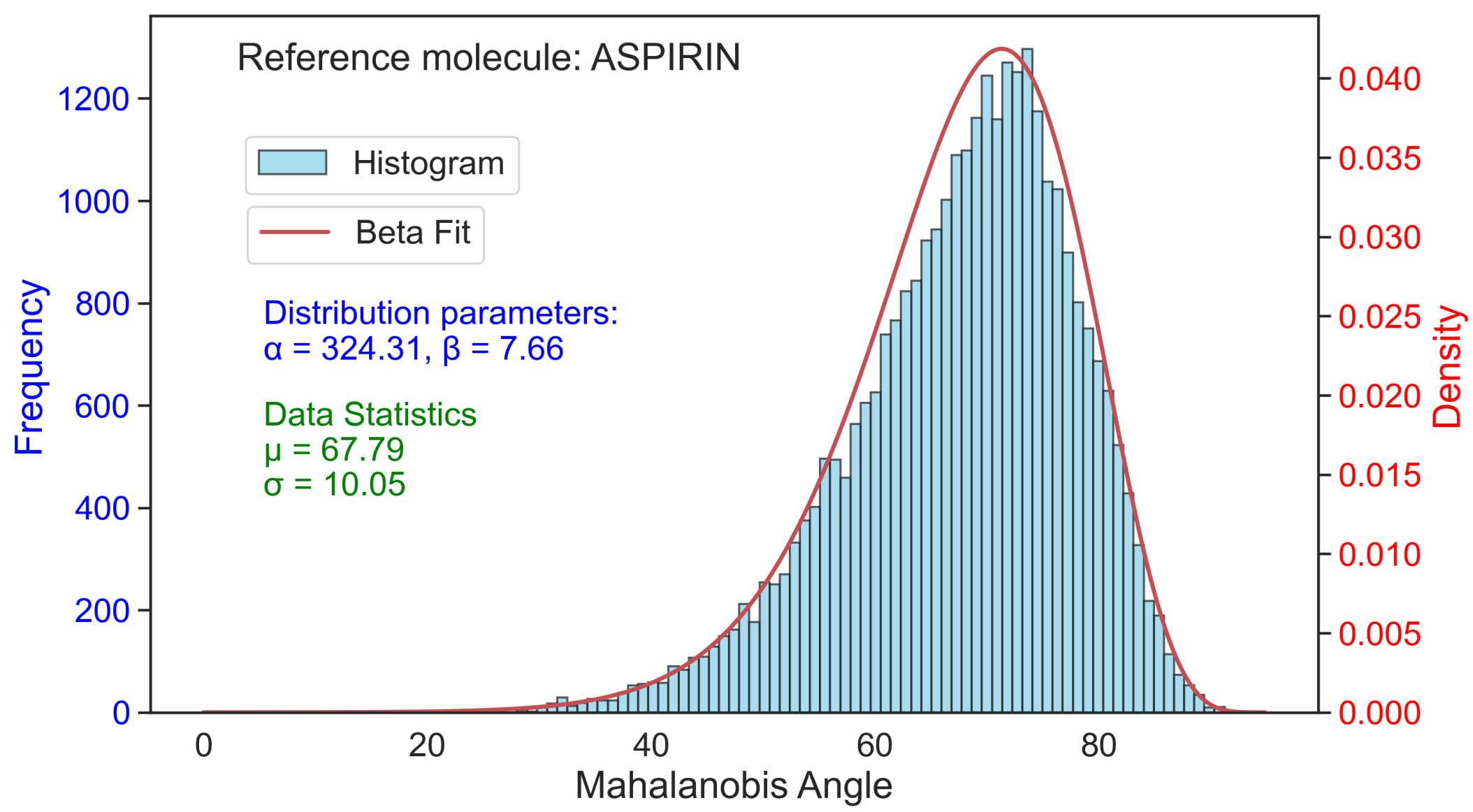

Figure S3. **Distribution of Mahalanobis angle values for the ACI dataset referenced to aspirin.** Blue bars represent the frequency histogram of Mahalanobis angle (θ_M) values across all molecules in the ACI library. The red curve shows the fitted beta distribution with shape parameters $\alpha = 324.31$ and $\beta = 7.66$. Data statistics indicate mean $\mu = 67.79$ and standard deviation $\sigma = 10.05$. The distribution exhibits a narrow, approximately symmetric peak centered at $\theta_M \approx 65$–$70°$, indicating strong directional alignment of most ACI molecules with aspirin in the Mol2Vec embedding space. The concentration within $\theta_M = 50$–$85°$ confirms that molecular variation occurs predominantly along similar descriptor directions rather than through angular dispersion, consistent with the chemically coherent nature of the aspirin-focused ACI library.

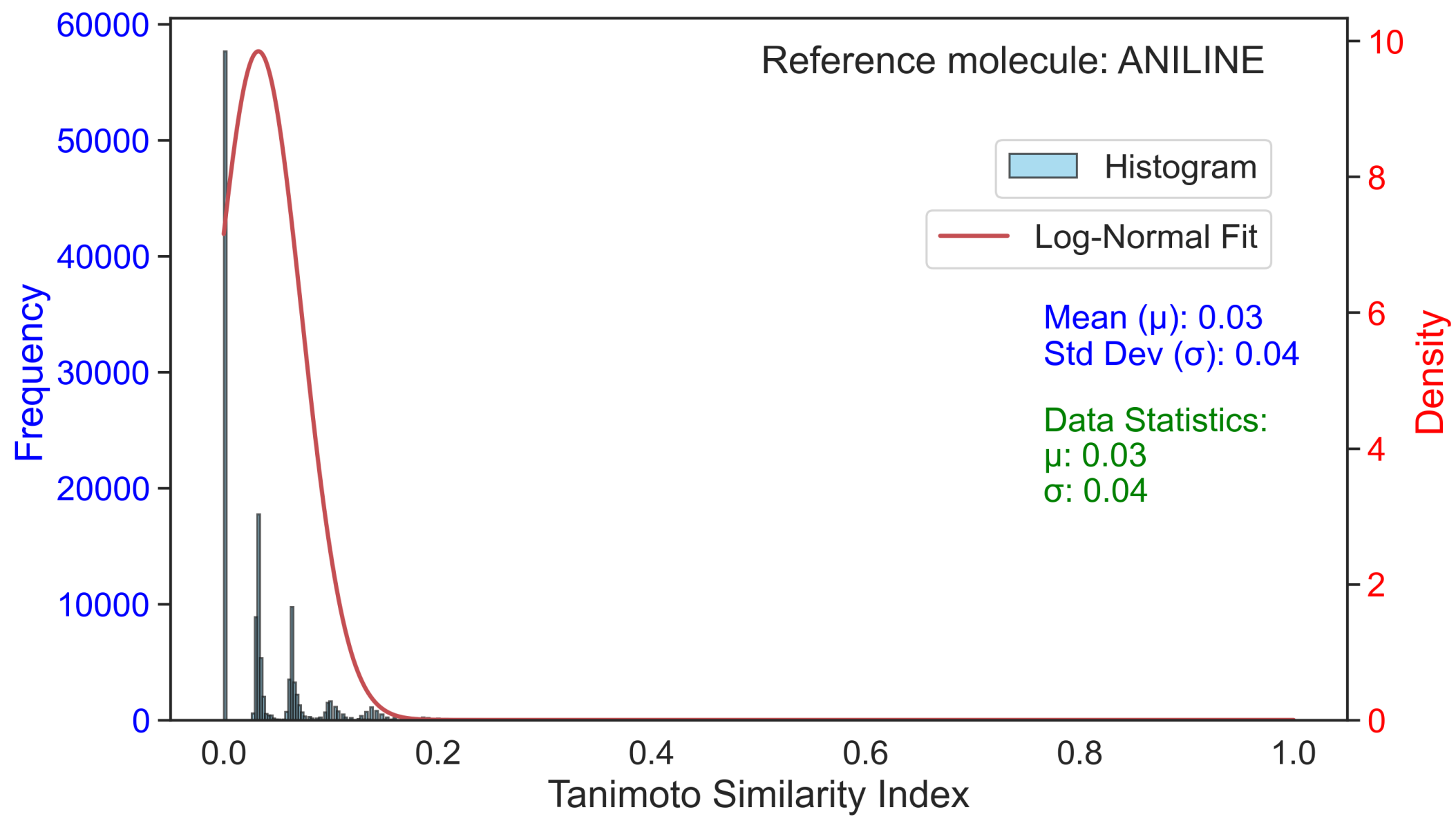

Figure S4. **Distribution of Tanimoto Similarity Index values for the QM9 dataset referenced to aniline.** Blue bars represent the frequency histogram of TSI values across all molecules in the QM9 database. The red curve shows the fitted log-normal distribution. Data statistics indicate mean $\mu = 0.03$ and standard deviation $\sigma = 0.04$. The distribution exhibits extreme positive skewness with maximum frequency at TSI ≈ 0.0, indicating that the vast majority of molecules in the QM9 dataset share minimal structural similarity with aniline. The sharp peak near zero and rapid decay reflect the chemical diversity of QM9, which encompasses a broad range of organic molecules beyond simple aromatic amines. Only a small fraction of molecules achieves TSI > 0.1, confirming aniline's specificity within this general-purpose molecular database.

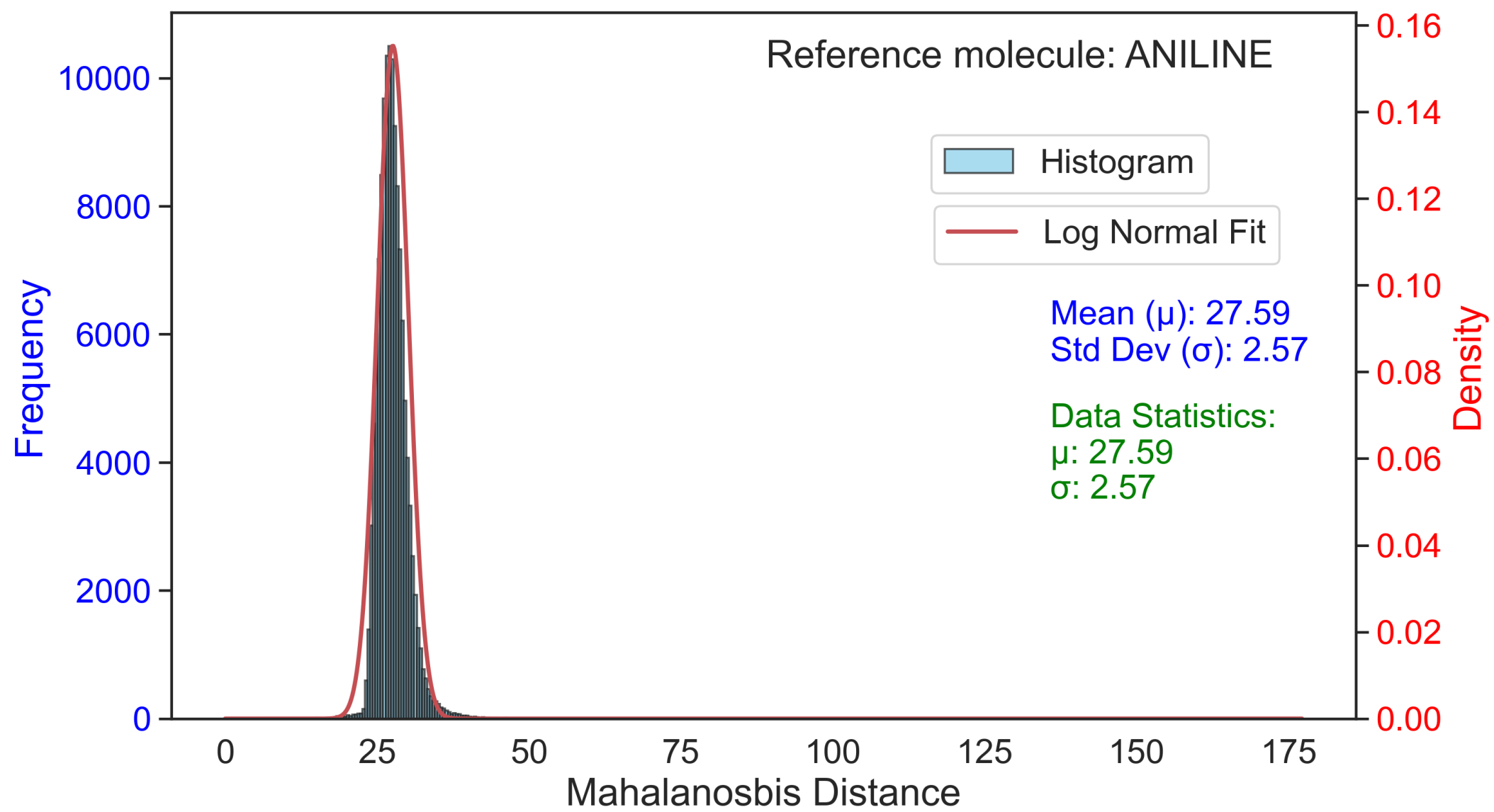

Figure S5. **Distribution of Mahalanobis distance values for the QM9 dataset referenced to aniline.** Blue bars represent the frequency histogram of Mahalanobis distance (d_M) values across all molecules in the QM9 database. The red curve shows the fitted log-normal distribution. Data statistics indicate mean $\mu = 27.59$ and standard deviation $\sigma = 2.57$. The distribution exhibits a sharp, narrow peak centered at $d_M \approx 27$, with minimal spread (σ only 9.3% of μ), indicating remarkably uniform Mahalanobis separation of QM9 molecules from aniline. This tight clustering contrasts sharply with the broader, more extended distribution observed for aspirin-ACI ($\mu = 15.33$, $\sigma = 8.58$), reflecting the systematic chemical diversity of QM9's general organic space versus the focused analog character of the aspirin library. The absence of molecules at low d_M values confirms that QM9 contains few close aniline analogs, consistent with the near-zero TSI distribution.

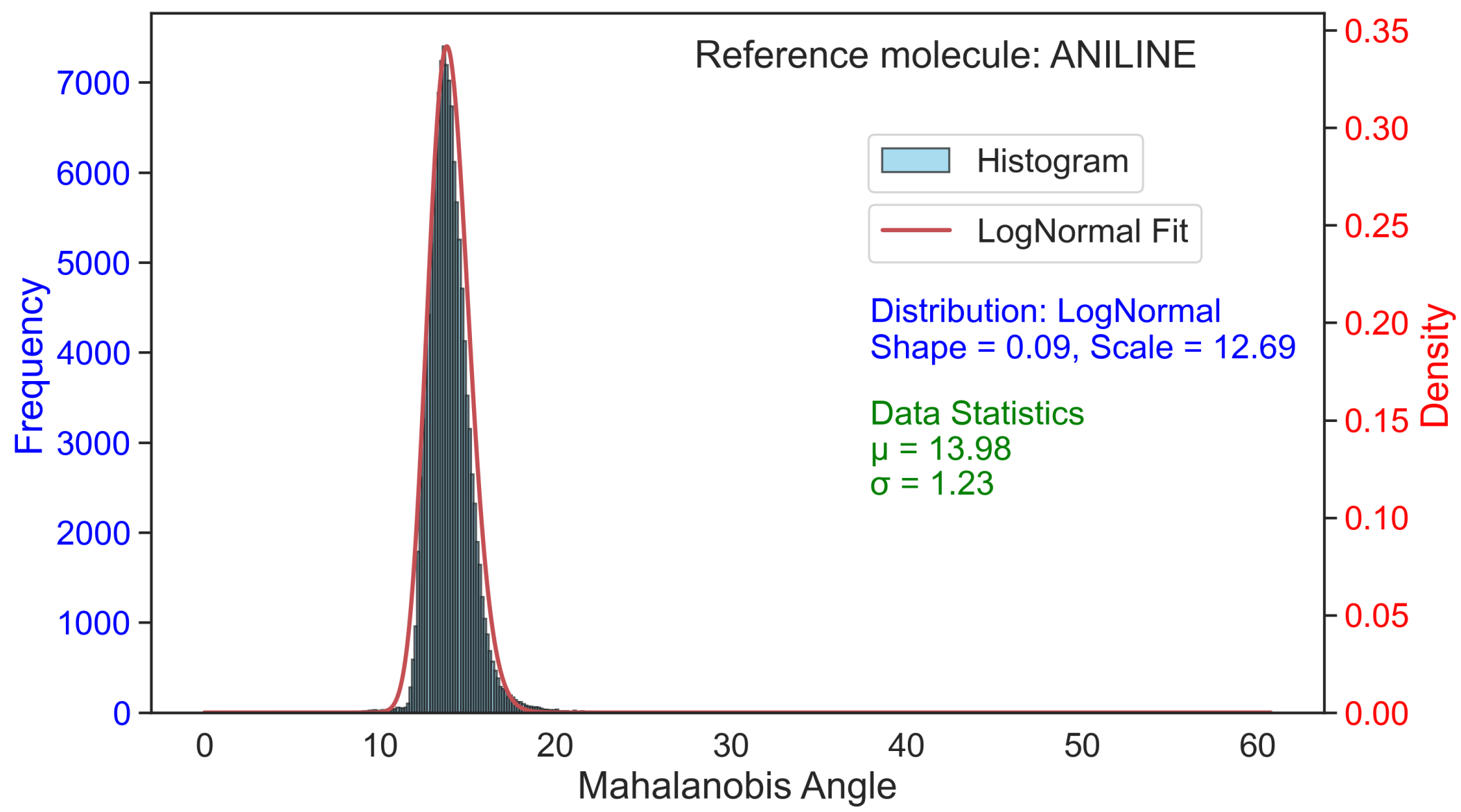

Figure S6. **Distribution of Mahalanobis distance values for the QM9 dataset referenced to aniline.** Blue bars represent the frequency histogram of Mahalanobis angle (θ_M) values across all molecules in the QM9 database. The red curve shows the fitted log-normal distribution with shape parameter = 0.09 and scale parameter = 12.69. Data statistics indicate mean $\mu = 13.98°$ and standard deviation $\sigma = 1.23°$. The distribution exhibits an extremely narrow, sharp peak centered at $\theta_M \approx 14°$, with minimal angular spread (coefficient of variation 8.8%), indicating remarkably uniform directional alignment of QM9 molecules relative to aniline in the Mol2Vec embedding space. This tight angular clustering at low θ_M values, combined with the broader distance distribution ($d_M \approx 27$), reveals anisotropic chemical space coverage: QM9 molecules maintain consistent directional orientation while varying primarily in radial separation. The concentration at $\theta_M < 20°$ places most QM9 entries within the angular applicability domain identified in the main text, enabling MSI to discriminate among molecules despite uniformly low TSI values.
